# DTX3 Suppresses the NPM3–RUVBL1 Axis to Inhibit Homologous Recombination Repair and Abrogate Tumor Radioresistance

**DOI:** 10.1002/advs.78573

**Published:** 2026-10-29

**Authors:** Jia‐Hao Xiao, Chang‐Jun Xie, Yan‐Hua Chen, Hao Cheng, Shuang‐Hui Li, Wei Li, Ya‐Wen Bin, Rui‐Min He, Ying‐Qing Li, Ping Feng

**Affiliations:** ^1^ Department of Cancer Center Hengyang Medical School The Second Affiliated Hospital University of South China Hengyang Hunan China; ^2^ Department of Breast and Thyroid Surgery Hengyang Medical School The Second Affiliated Hospital University of South China Hengyang Hunan China; ^3^ State Key Laboratory of Oncology in South China Guangdong Key Laboratory of Nasopharyngeal Carcinoma Diagnosis and Therapy Guangdong Provincial Clinical Research Center for Cancer Sun Yat‐sen University Cancer Center Guangzhou China

**Keywords:** deltex 3, homologous recombination repair, nucleophosmin 3, tumor radioresistance, ubiquitination

## Abstract

Aberrant remodeling of DNA damage repair pathways after radiotherapy constitutes a primary driver of tumor radioresistance and compromised therapeutic efficacy. Ubiquitination has been reported as a critical post‐translational modification governing the DNA damage response and repair cascade. However, the precise molecular mechanisms underlying ubiquitination‐mediated radioresistance remain poorly characterized. The E3 ubiquitin ligase Deltex 3 (DTX3) is validated as a negative regulator of tumor radioresistance that improves radiotherapy efficacy in vitro and in vivo models. Mechanistically, DTX3 physically interacts with nucleophosmin 3 (NPM3), catalyzes K27‐linked polyubiquitination of NPM3 at the Lys102 residue, and consequently triggers proteasomal degradation of NPM3. Loss of NPM3 further destabilizes the RUVBL1–TIP60 complex, diminishes H4K16 acetylation, and impairs homologous recombination repair. Consistently, elevated NPM3 expression counteracts DTX3‐mediated radiosensitization. Clinically, low DTX3 levels are correlated with inferior overall survival and elevated post‐radiotherapy recurrence across multiple human malignancies. The study reveals DTX3 as a potent suppressor of homologous recombination repair and a promising therapeutic candidate to overcome tumor radioresistance.

## Graphical abstract

Deltex 3 (DTX3) catalyzes K27‐linked ubiquitination of nucleophosmin 3 to disrupt homologous recombination repair and reverse tumor radioresistance. Low DTX3 predicts poor survival and radiotherapy recurrence, serving as a radiosensitizing therapeutic target.

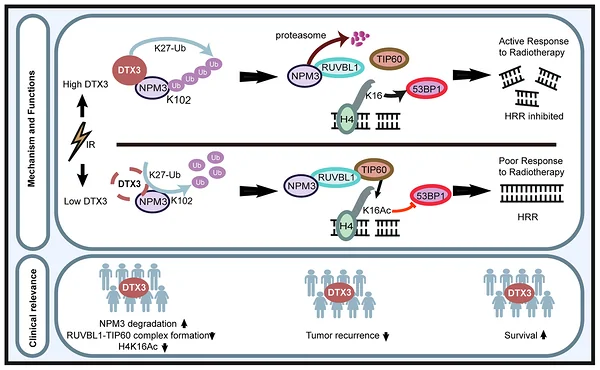

## 1 Introduction

Radiotherapy (RT) is a cornerstone of cancer treatment and the second most common therapeutic modality after surgery. Despite its widespread use, radioresistance remains a major clinical challenge and a key determinant of therapeutic efficacy. DNA damage repair (DDR) pathways regulate tumor radiosensitivity and are central to the development of radioresistance [1]. Radiation‐induced DNA double‐strand breaks (DSBs) are primarily repaired through nonhomologous end joining (NHEJ) and homologous recombination repair (HRR), with NHEJ being error‐prone and HRR providing high fidelity [2]. Dysregulated expression of factors involved in NHEJ or HRR can confer radioresistance and promote tumor progression [3, 4]. Therefore, targeting DDR signaling pathways has emerged as a promising strategy to overcome tumor radioresistance [5].

The ubiquitin–proteasome system (UPS) is a dynamic regulatory network that controls protein stability and function through coordinated ubiquitination and deubiquitination. As a major post‐translational modification system, the UPS regulates protein turnover and signaling pathways, thereby influencing DNA repair, metabolic reprogramming, immune evasion, and cellular stress responses. These processes collectively modulate tumor radiosensitivity [6]. For example, USP7 deubiquitinates and stabilizes TREX1, leading to excessive clearance of cytosolic double‐stranded DNA and attenuation of the immune response, thereby promoting radioresistance [7]. USP18 regulates the TRIM21–TRIM29 axis via ubiquitination to enhance radioresistance in nasopharyngeal carcinoma (NPC) [3]. In esophageal squamous cell carcinoma, NEDD4L‐mediated ubiquitination of KLF5 suppresses radioresistance [8]. In addition, the E3 ligase TRIM21 promotes ubiquitination and degradation of MLPH, thereby reducing radioresistance in glioblastomas [9]. We previously demonstrated that BRCC3 deubiquitinates and stabilizes PPP1CC, activates DNA‐PKcs, and enhances radiation‐induced NHEJ repair, thereby increasing radioresistance in NPC [10]. Collectively, these findings highlight the central role of ubiquitination and deubiquitination in regulating radiosensitivity. Targeting components of the UPS may therefore provide novel strategies to overcome tumor radioresistance [11]. However, the specific roles and mechanisms of E3 ligases in DDR signaling and radioresistance remain incompletely defined.

The human Deltex protein family comprises five members with E3 ubiquitin ligase activity: DTX1, DTX2, DTX3, DTX3L, and DTX4. To date, studies investigating the involvement of the Deltex family in DNA repair remain limited, with most research focusing on DTX2 and DTX3L. For example, DTX2 recognizes and recruits ADP‐ribosylated proteins through its C‐terminal domain (CTD) and ubiquitinates proteins involved in DDR, thereby modulating the DDR process [12]. A recent study by Djerir et al. further demonstrated that DTX2 is recruited to DSB sites and stably retained there through its WWE domain and conserved C‐terminal Deltex domain, where it participates in HRR‐dependent DSB repair [13]. DTX3L, another member of the Deltex family, monoubiquitinates histone H4 at lysine 91, thereby facilitating the recruitment of 53BP1, and enhancing cellular sensitivity to DNA‐damaging agents [14]. Overexpression of DTX3L promotes the ubiquitination and proteasomal degradation of Tudor interacting repair regulator (TIRR), thereby abolishing the inhibitory effect of TIRR on 53BP1. This process reduces HRR efficiency and renders prostate cancer cells susceptible to poly (ADP‐ribose) polymerase (PARP) inhibitors [15]. Moreover, DTX3L interacts with BAL1 and recruits BRCA1 in a PARP1‐dependent manner to regulate DSB repair [16]. Collectively, these findings indicate that Deltex family proteins, particularly DTX2 and DTX3L, play important roles in the DDR pathway and DSB repair. Nevertheless, no previous studies have reported a functional role for DTX3 in DDR.

In this study, by analyzing RNA sequencing data, we screened Deltex 3 (DTX3) as an E3 ligase that may reverse radioresistance. Further functional experiments confirmed that the overexpression of DTX3 reversed the radioresistance of tumor cells. Mechanistically, DTX3 mediates the ubiquitination and degradation of nucleophosmin 3 (NPM3), thereby disrupting the formation of the RuvB‐like protein 1‐lysine acetyltransferase (RUVBL1–TIP60) complex, reducing histone H4K16 acetylation, and impairing HRR. Clinically, low DTX3 expression is associated with poor RT‐related prognosis. These findings highlight the critical role of DTX3 in DDR signaling and radiosensitivity and identify it as a potential therapeutic target to overcome tumor radioresistance.

## 2 Results

### 2.1 Screening and Identification of DTX3 as a Gene that Reverses Radioresistance

To identify E3 ubiquitin ligases that may be responsible for reversing radioresistance, we screened two independent data sets: (1) TCGA‐head and neck squamous cell carcinoma (HNSC), comprising 121 patients with RT follow‐up information (no locoregional recurrence: n = 106, locoregional recurrence: n = 15) and (2) GSE185421, comprising NPC cell lines with radiosensitivity data. Differential expression analyses were performed using stringent thresholds in both datasets. The results revealed an overlap in downregulated genes between locoregional recurrent HNSC tissues and radioresistant cell lines (Figure 1A). In the TCGA‐HNSC dataset, using thresholds of |FoldChange| > 1.5 and p < 0.01, the recurrent group exhibited 942 downregulated and 579 upregulated genes compared with the nonrecurrent group. In the GSE185421 dataset, using thresholds of |FoldChange| > 2.0 and adjusted p < 0.01, the radioresistant cell CNE2‐IR exhibited 230 downregulated and 251 upregulated genes compared with the radiosensitive CNE2 cells (Figure 1B). Subsequently, these downregulated genes were intersected with the reported E3 ligase library genes of the human genome [17] (Table S1). Only DTX3 remained (Figure 1C). Given that the role and mechanism of DTX3 in tumor radioresistance have not been studied, our subsequent investigation focused on DTX3.

**FIGURE 1:**
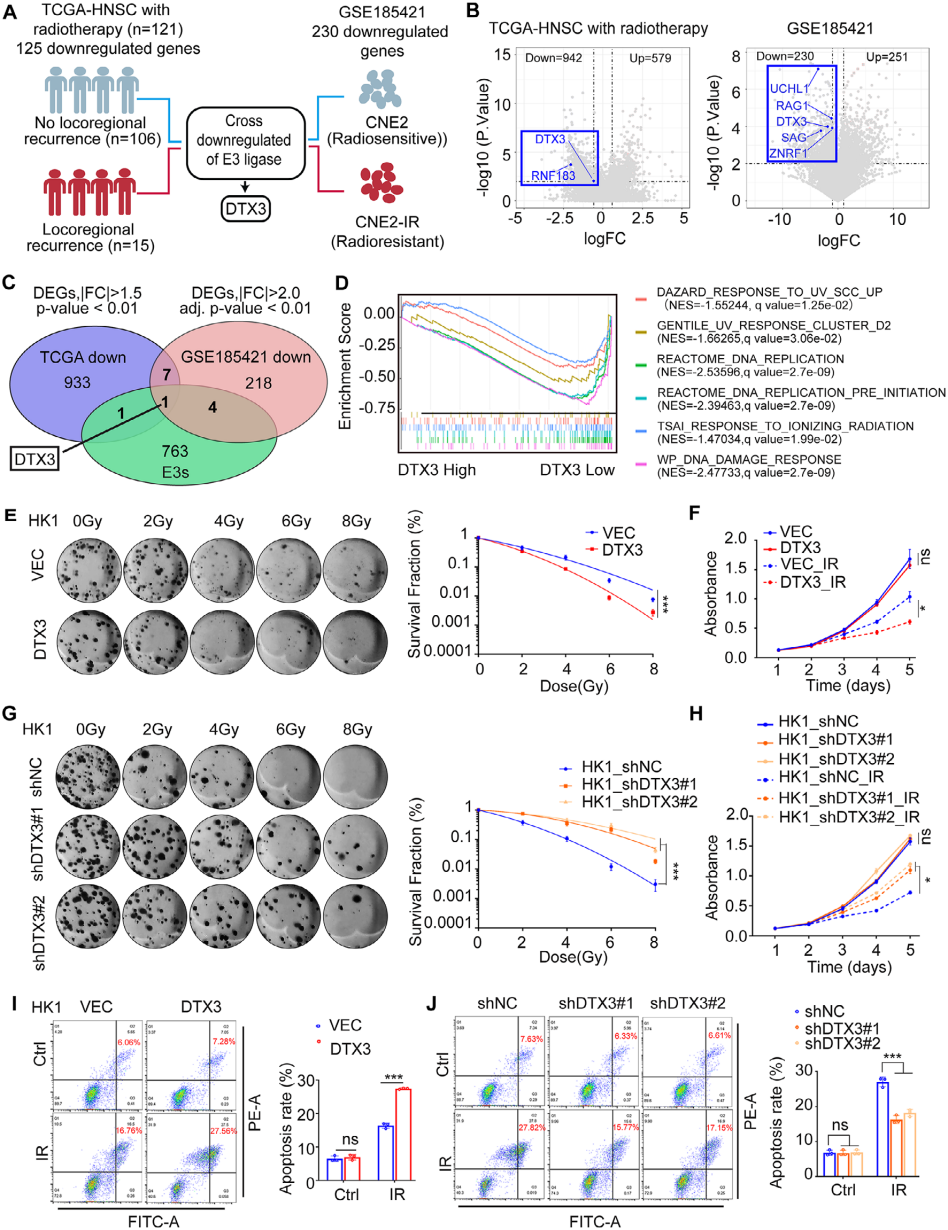
Screening and identification of DTX3 as a gene that reverses radioresistance. (A) Schematic diagram showing the screening process of DTX3 from TCGA‐HNSC and GSE185421. (B) Volcano plot showing the differentially expressed genes of TCGA‐HNSC and GSE185421. (C) The Venn diagram shows overlapping downregulated genes identified across three datasets: TCGA‐HNSC, GSE185421, and the E3 library database. (D) Gene set enrichment analysis (GSEA) of the GSE12452 dataset revealed enrichment of UV response and DNA damage response pathways in the DTX3 low‐expression group. (E,F) HK1 cells with or without DTX3 overexpression were exposed to the indicated dose of irradiation for clonogenic survival assays (E) and to 6 Gy irradiation for Cell Counting Kit‐8 (CCK‐8) viability assays (F). Data are presented as the mean ± SD (n = 3; extra sum‐of‐squares F test was used for **E**, two‐way ANOVA was used for **F**; ^*^ *p* < 0.05, ^***^ *p* < 0.001). (G,H) HK1 cells with or without DTX3 knockdown were exposed to irradiation for clonogenic survival assays (G) and to 6 Gy irradiation for CCK‐8 assays (H). Data are presented as the mean ± SD (n = 3; extra sum‐of‐squares F test was used for G, two‐way ANOVA was used for H; ^*^ *p* < 0.05, ^***^ *p* < 0.001). (I,J) Flow cytometric analysis of apoptosis in HK1 cells with DTX3 overexpression (I) or knockdown (J) at 24 h after exposure to either no irradiation or 6 Gy irradiation. Data are presented as the mean ± SD (n = 3; unpaired t‐test was used for I, one‐way ANOVA was used for J; ^***^ *p* < 0.001). n = 3 independent experiments. Abbreviations: IR, irradiation; HNSC, head and neck squamous cell carcinoma.

Gene set enrichment analysis (GSEA) suggested that the high expression of DTX3 was negatively correlated with pathways related to radiation response and DDR (Figure 1D). To further investigate the effect of DTX3 on tumor cell radiosensitivity, we constructed HK1 (human NPC) and HeLa (human cervical cancer) cell lines stably overexpressing DTX3 or knocking down DTX3 (sh*DTX3*) (Figure S1A–D). Both cell lines are classic models for radiosensitivity research [18]. In both HK1 and HeLa cell lines, DTX3 overexpression significantly reduced colony formation after irradiation (*p* < 0.001; extra sum‐of‐squares F test; Figure 1E, Figure S1E). The surviving fraction at 2 Gy (SF_2_) decreased from 0.47 to 0.34 in HK1, yielding a sensitization enhancement ratio (SER) of 1.38 (0.47/0.34), while the SER reached 1.69 (0.54/0.32) in HeLa. CCK‐8 assays confirmed that DTX3 overexpression enhanced radiation‐induced growth inhibition (p = 0.039, HK1; p = 0.030, HeLa; two‐way ANOVA; Figure 1F, Figure S1F). Conversely, DTX3 knockdown promoted colony formation post‐irradiation, with SER values < 1 in both cell lines (*p* < 0.001; Figure 1G, Figure S1G). Similarly, CCK‐8 assays showed that DTX3 knockdown significantly attenuated radiation‐induced suppression of cell growth (*p* = 0.034, HK1; *p* = 0.010, HeLa; Figure 1H, Figure S1H). Irradiation‐induced DNA damage triggers robust apoptotic responses [19]. Our data showed that the combination of irradiation and DTX3 overexpression markedly increased tumor cell apoptosis, with apoptosis rates approximately 1.67‐fold and 1.77‐fold higher in HK1 and HeLa cells, respectively, than in the corresponding irradiation‐treated vector control groups (*p* < 0.001; unpaired t‐test; Figure 1I, Figure S1I). Conversely, DTX3 knockdown in irradiated tumor cells significantly attenuated apoptosis (*p* < 0.001; one‐way ANOVA; Figure 1J, Figure S1J). Together, these findings indicated that DTX3 reduces the radioresistance of tumor cells by inhibiting proliferation and promoting apoptosis.

### 2.2 DTX3 Impairs DSB Repair by Suppressing the HRR Repair Pathway

The role of DTX3 in DDR was characterized in HK1 and HeLa cells, comparing conditions with and without DTX3 overexpression using a neutral comet assay to detect DSBs. In both cell lines, DTX3 overexpression significantly delayed DNA repair, as indicated by the longer tail moments in the irradiated cells (*p* < 0.001; two‐way ANOVA; Figure 2A, Figure S2A). The mean tail moment at 24 h after irradiation was 1.64‐fold higher in DTX3‐overexpressing HK1 cells (35.64 ± 5.90%) and 1.94‐fold higher in DTX3‐overexpressing HeLa cells (37.01 ± 7.70%) than in the corresponding control cells (HK1: 21.69 ± 5.51%; HeLa: 19.07 ± 7.23%). Irradiation induces DSBs, accompanied by rapid phosphorylation of histone H2AX at serine 139 (γ‐H2AX) at damage sites [20]. Following irradiation, γ‐H2AX levels were assessed by western blotting and immunofluorescence in DTX3‐overexpressing and control cells. In DTX3‐overexpressing cells, γ‐H2AX accumulation was markedly increased (Figure 2B, Figure S2B), and the number of γ‐H2AX foci remained elevated for up to 24 h (*p* < 0.001; two‐way ANOVA; Figure 2C, Figure S2C). These results suggested that DTX3 inhibits DSB repair.

**FIGURE 2:**
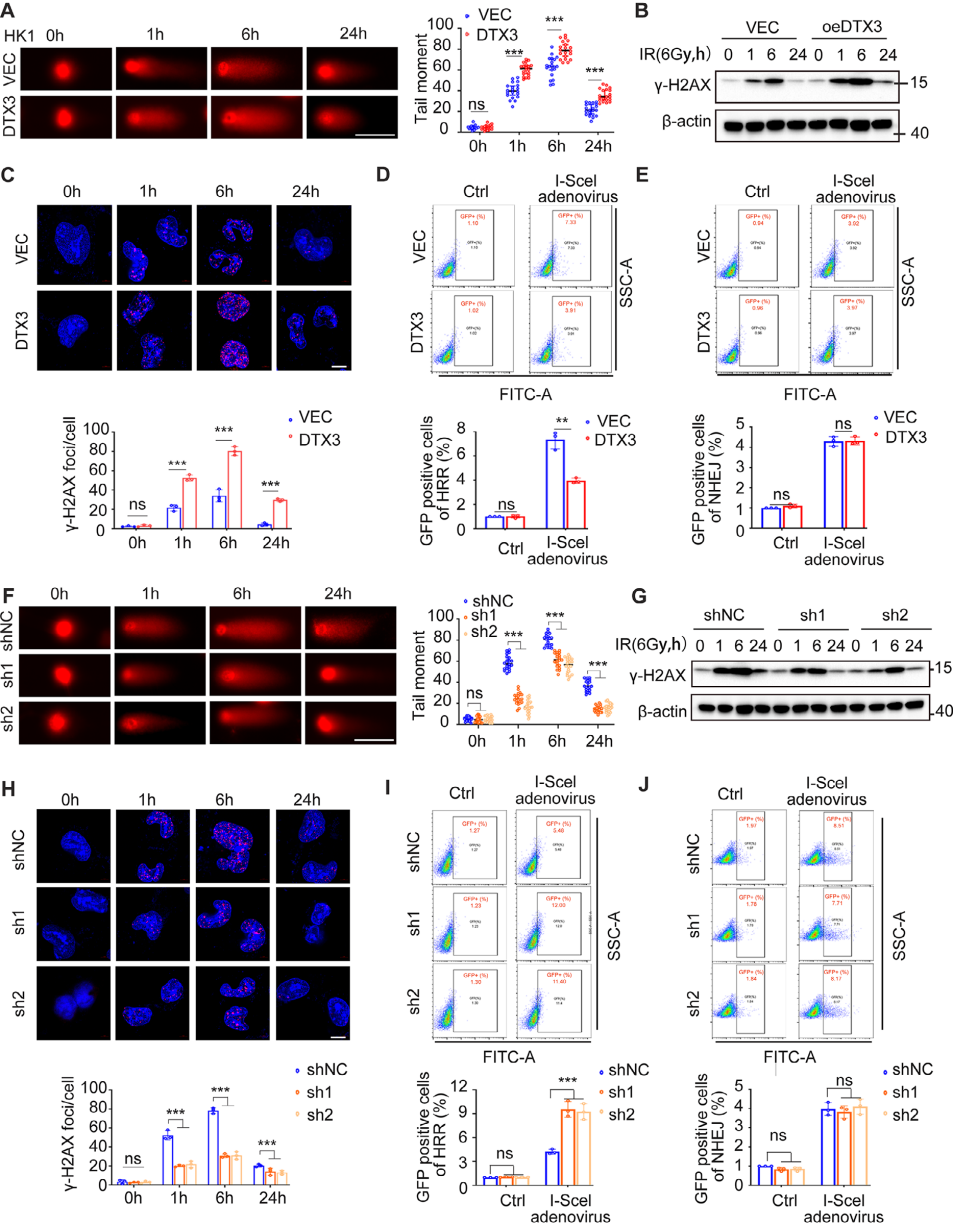
DTX3 impairs DNA double‐strand break (DSB) repair by inhibiting the homologous recombination repair (HRR) pathway. (A) HK1 cells with or without DTX3 overexpression were collected at the indicated time points after 6 Gy irradiation for the neutral comet assay. Tail moment was analyzed using CaspLab‐Comet Assay software, scale bar: 50 µm. Data are presented as the mean ± SD (n = 20; two‐way ANOVA; ^***^ *p* < 0.001). The experiments were repeated three times. (B) The expression of γ‐H2AX in HK1 cells with or without DTX3 overexpression after irradiation was analyzed by western blotting. (C) Representative fluorescence images and quantification of γ‐H2AX foci in HK1 cells with or without DTX3 overexpression. Above: representative fluorescence images (scale bars, 10 µm; red, γ‐H2AX; blue, DAPI). Under: percentage of positive cells with γ‐H2AX foci (> 10). Each datum point contains at least 20 cells. Data are presented as the mean ± SD (n = 3 independent experiments; two‐way ANOVA; ^***^ *p* < 0.001). (D,E). Characterization of DSB repair pathways using HRR (D) and non‐homologous end joining (NHEJ) (E) reporter assays. The proportion of GFP^+^ cells was measured by flow cytometry and the efficiency was normalized to the control cells. Data are presented as mean ± SD (n = 3 independent experiments; unpaired t‐test; ns, not significant; ^**^ *p* < 0.01). (F). HK1 cells with or without DTX3 knockdown were collected at the indicated time points after 6 Gy irradiation for the neutral comet assay. Tail moment was analyzed with CaspLab‐Comet Assay software, scale bar: 50 µm. Data are presented as the mean ± SD (n = 20; two‐way ANOVA; ^***^ *p* < 0.001). The experiments were repeated three times. (G) The expression of γ‐H2AX in DTX3 knockdown or not cells after irradiation was analyzed by western blotting. (H) Representative fluorescence images and quantification of γ‐H2AX foci in HK1 cells with or without DTX3 knockdown. Above: representative fluorescence images (scale bars, 10 µm; red, γ‐H2AX; blue, DAPI). Under: percentage of positive cells with γ‐H2AX foci (>10). Each datum point contains at least 20 cells. Data are presented as the mean ± SD (n = 3 independent experiments; two‐way ANOVA; ^***^ *p* < 0.001). (I,J) Characterization of DSB repair pathways using HRR (I) and NHEJ (J) reporter assays. The proportion of GFP^+^ cells was measured by flow cytometry and the efficiency was normalized to the control cells. Data are presented as mean ± SD (n = 3 independent experiments; one‐way ANOVA; ns, not significant; ^***^ *p* < 0.001). n = 3 independent experiments. Abbreviations: IR, irradiation. The unprocessed images of the blots are shown in Figure S23.

To identify the DSB repair pathway regulated by DTX3, two reporter assays were used to quantify NHEJ and HRR activity (Figure S1K,L). DTX3 overexpression did not alter NHEJ efficiency but significantly reduced HRR efficiency by 46% in irradiated HK1 cells (*p* = 0.003), and by 28% in irradiated HeLa cells (*p* = 0.001; unpaired *t*‐test; Figure 2D,E, Figure S2D,E). These findings suggested that DTX3 inhibits DSB repair by inhibiting the HRR pathway.

Finally, we confirmed that DTX3 knockdown promotes DDR in HK1 and HeLa cells. Irradiation‐induced tail moments were shortened in DTX3 knockdown cells (*p* < 0.001; two‐way ANOVA; Figure 2F, Figure S2F). These cells also exhibited lower γ‐H2AX levels and fewer γ‐H2AX foci (*p* < 0.001; two‐way ANOVA; Figure 2G,H, Figure S2G,H). HRR efficiency increased following DTX3 knockdown, whereas NHEJ activity remained unaffected under irradiation (*p* < 0.001; one‐way ANOVA; Figure 2I,J, Figure S2I–J). Overall, these results indicated that DTX3 functions as a negative regulator of DDR primarily by suppressing HRR‐mediated DSB repair.

To confirm the targeting specificity of sh*DTX3*, rescue experiments were performed (Figure S3A). Re‐expression of DTX3 in sh*DTX3* HK1 and HeLa cells reversed the phenotypes induced by DTX3 knockdown, including increased clonogenic survival (Figure S3B,C), suppressed apoptosis (Figure S4), and enhanced HRR efficiency (Figure S5).

### 2.3 DTX3 Interacts with NPM3 and Negatively Regulates NPM3 Protein Expression

To investigate the mechanism by which DTX3 modulates tumor cell radiosensitivity, HK1 cells overexpressing HA‐tagged DTX3 were subjected to immunoprecipitation (IP) with an anti‐HA antibody. Co‐IP proteins were separated via sodium dodecyl sulfate‐polyacrylamide gel electrophoresis (SDS‐PAGE), and the bands corresponding to approximately 180 and 25 kDa were excised for mass spectrometry (MS) analysis (Figure 3A). The MS data are presented in Table S2. Based on bioinformatic analyses and molecular weight considerations, the potential DTX3 interactors included TOP2A, TOP2B, PSMB2, and NPM3 (Figure S6A). Subsequent IP and western blotting identified NPM3, but not TOP2A, TOP2B, or PSMB2, as interacting with DTX3 (Figure 3B). The exogenous and endogenous DTX3–NPM3 interactions were further confirmed by Co‐IP (Figure 3C, Figure S6B,C). An in vitro GST pull‐down assay revealed a direct interaction between DTX3 and NPM3 (Figure 3D). Moreover, this association was not significantly altered over 24 h after irradiation (Figure 3E, Figure S6D). DTX3 and NPM3 colocalized in the nucleus following irradiation (Figure 3F, Figure S6E). These findings demonstrated that DTX3 directly interacts with NPM3.

**FIGURE 3:**
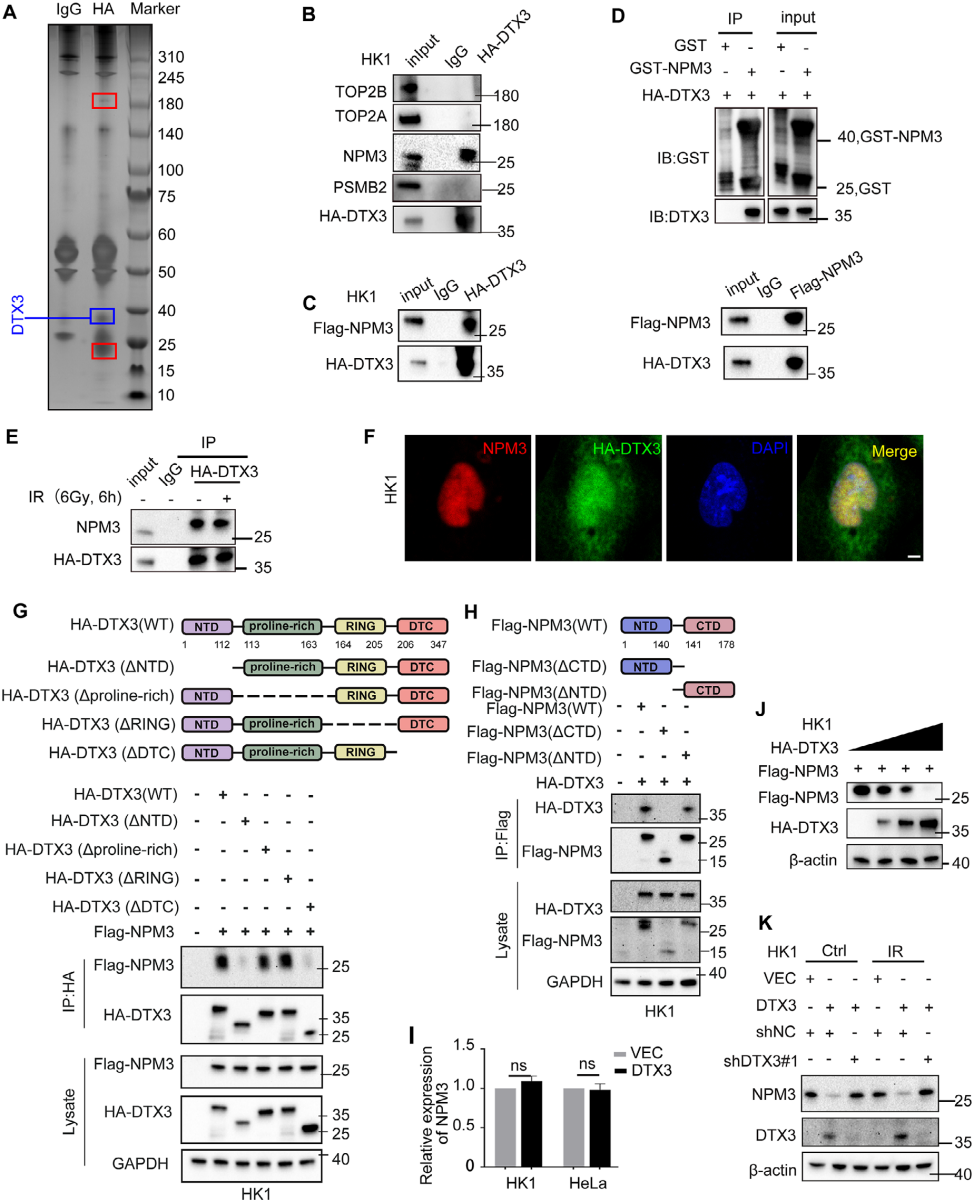
DTX3 interacts with NPM3 and negatively regulates NPM3 protein expression. (A) HA‐immunoprecipitated proteins separated by SDS‐PAGE from HK1 cells stably overexpressing HA‐DTX3. Bands indicated in the red boxes were cut and subjected to mass spectrometry analysis. (B) Western blot analyses of proteins co‐immunoprecipitated (Co‐IP) with an HA antibody for expression of the indicated proteins. (C) DTX3–NPM3 exogenous interaction detected by Co‐IP in HK1 cells coexpressing HA‐DTX3 and Flag‐NPM3. (D) GST pull‐down assay indicating the direct interaction of purified GST‐NPM3 protein with HA‐DTX3 protein. (E) The association of HA‐DTX3 and NPM3 was detected by Co‐IP in HK1 cells with or without irradiation treatment. (F) Immunofluorescence staining showed nuclear colocalization of exogenous HA‐DTX3 (green) and endogenous NPM3 (red) at 6 h following exposure to 6 Gy in HK1 cells. Scale bars: 5 µm. (G) Schematic representation of the full length of DTX3 and its truncated variants (top). Co‐IP and immunoblot analyses of lysates from HK1 cells transfected with HA‐DTX3 or its mutants and Flag‐NPM3. (H) Schematic representation of the full length of NPM3 and its truncated variants (top). Co‐IP and immunoblot analyses of lysates from HK1 cells transfected with Flag‐NPM3 or its mutants and HA‐DTX3. (I,J) DTX3 did not affect *NPM3* mRNA expression (I) but reduced NPM3 protein expression (J) in the indicated cells. (K) HK1 cells were transfected with *DTX3, vector, shNC or shDTX3#1* to detect the expressions of NPM3 after irradiation with 6 Gy for 6 h. Data in I are presented as the mean ± SD, n = 3 independent experiments. Abbreviations: IR, irradiation. The unprocessed images of the blots are shown in Figure S23.

To further identify the specific domains of DTX3 responsible for its interaction with NPM3, a series of DTX3 deletion mutants was generated (Figure 3G). Co‐IP assays showed that deletions of either the N‐terminal domain (NTD) or the Deltex carboxyl‐terminal (DTC) domain of DTX3 disrupted its interaction with NPM3 (Figure 3G, Figure S6F), indicating that both domains are required for this interaction. To identify the NPM3 domains mediating its interaction with DTX3, several Flag‐tagged NPM3 truncation mutants were constructed (Figure 3H). Co‐IP assays revealed that the deletion of CTD abolished the interaction between NPM3 and DTX3 (Figure 3H, Figure S6G). These results clearly demonstrate that the NPM3 CTD domain is required for its interaction with DTX3.

Next, we investigated whether DTX3 regulates the expression of NPM3. The results showed that DTX3 overexpression downregulated NPM3 protein expression in a dose‐dependent manner without altering NPM3 mRNA levels (Figure 3I,J). Conversely, DTX3 knockdown largely reversed this decrease and showed no association with DNA damage (Figure 3K, Figure S6H). These results suggested that DTX3 could negatively regulate NPM3 protein expression.

### 2.4 DTX3 Mediates K27‐Linked Polyubiquitination of NPM3 at Lysine Residue 102 and Promotes its Degradation

DTX3 is an E3 ligase that ubiquitinates substrates for proteasomal degradation, suggesting that it may target NPM3 for degradation. Following cycloheximide (CHX) treatment, DTX3 expression accelerated NPM3 degradation, whereas DTX3 knockdown attenuated degradation, indicating that DTX3 shortens the half‐life of NPM3 protein (*p* < 0.01; two‐way ANOVA; Figure 4A,B, Figure S7A,B). Protein degradation occurs mainly through the ubiquitin–proteasome and lysosomal pathways. Coexpression of DTX3 and NPM3 in the presence of the proteasome inhibitor MG132 or the lysosomal inhibitor chloroquine (CQ) showed that MG132 blocked DTX3‐mediated NPM3 degradation, whereas CQ had no effect (Figure 4C, Figure S7C). DTX3 knockdown increased NPM3 expression, and MG132 reversed this effect, whereas CQ had no effect (Figure 4D, Figure S7D). We also assessed endogenous NPM3 levels in DTX3‐overexpressing cells treated with the MG132. The results revealed that DTX3 suppresses NPM3 expression through the ubiquitin‐proteasome pathway, and that this regulatory effect remained unchanged after irradiation (Figure 4E, Figure S7E). In summary, DTX3 promotes NPM3 degradation via the ubiquitin–proteasome pathway.

**FIGURE 4:**
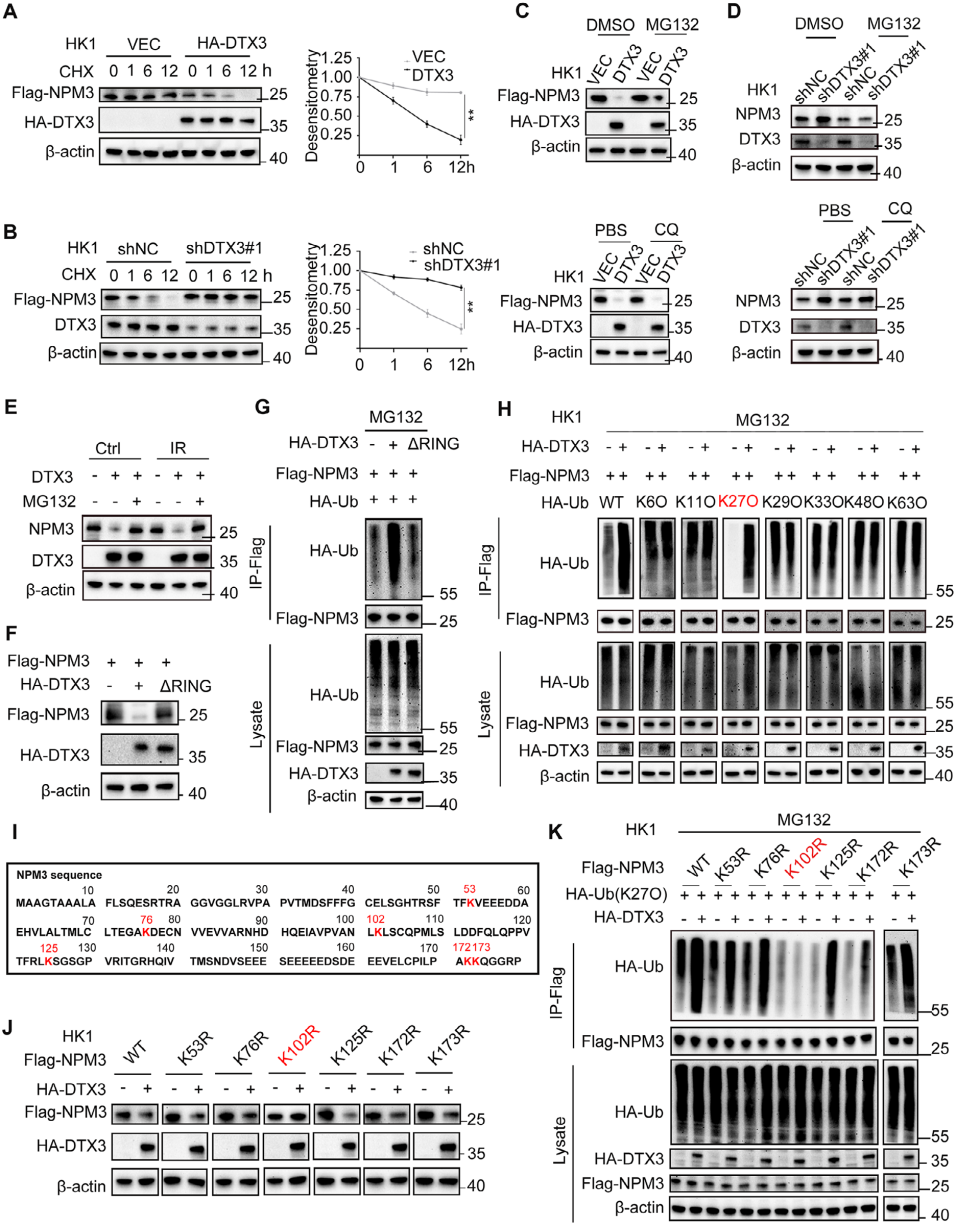
DTX3 mediates K27‐linked polyubiquitination of NPM3 at lysine residue 102 and promotes its degradation. (A,B) Effects of cycloheximide (CHX) treatment in cells transfected with Flag‐*NPM3* and HA‐*DTX3* or empty vector plasmids (A), as well as in shNC or sh*DTX3#1* (B) in HK1 cells. Data are presented as the mean ± SD (n = 3 independent experiments; two‐way ANOVA; **p < 0.01). (C,D) The results of treatment with MG132 and chloroquine (CQ) in cells transfected with Flag‐*NPM3* and HA‐*DTX3* or empty vector plasmids (C), as well as in shNC or sh*DTX3#1* (D) in HK1 cells. (E) HK1 cells transfected with *DTX3* or vector were exposed to MG132 for 6 h prior to western blot analyses. (F) HK1 cells expressing Flag‐*NPM3* were transfected with vector, HA‐*DTX3(WT)* or HA‐*DTX3 (ΔRING)* and analyzed by western blotting. (G) Denaturing immunoprecipitation with anti‐Flag was performed in HK1 cells expressing the vector, HA‐*DTX3* or HA‐*DTX3 (ΔRING)* co‐transfected with Flag‐*NPM3* and HA‐*Ub*, followed by western blotting. (H) HK1 cells expressing HA‐*DTX3* or the empty vector were co‐transfected with Flag‐*NPM3* and HA‐*WT‐Ub* or its mutants (HA‐*K6O‐Ub*, HA‐*K11O‐Ub*, HA‐*K27O‐Ub*, HA‐*K29O‐Ub*, HA‐*K33O‐Ub*, HA‐*K48O‐Ub* or HA‐*K63O‐Ub*), and were treated with MG132. Western blotting images of denature‐IP experiments. (I) All the lysine sites of the NPM3 protein. (J) HK1 cells were co‐expressed with the indicated mutant Flag‐*NPM3* and HA‐*DTX3* or a control vector for 48 h before western blot analyses. (K). Denature‐IP was performed in HK1 cells expressing HA‐*DTX3* or vector after co‐transfection with HA‐*Ub(K27O)*, and Flag‐*NPM3* WT or KR mutants. n = 3 independent experiments. Abbreviations: IR, irradiation. The unprocessed images of the blots are shown in Figure S23.

The RING domain is essential for DTX3 E3 ligase activity [21, 22]. To investigate whether DTX3‐mediated ubiquitination drives NPM3 degradation, we ectopically expressed DTX3 or DTX3 ΔRING in HK1 cells. The results showed that the DTX3‐dependent ubiquitination of NPM3 required intact E3 ligase activity (Figure 4F,G).

Next, we identified the ubiquitin chain attachment sites on NPM3 promoted by DTX3. DTX3 overexpression increased NPM3 polyubiquitination, whereas DTX3 knockdown decreased it. Specifically, DTX3 overexpression enhanced the K27‐linked ubiquitination of NPM3, and DTX3 knockdown markedly reduced this ubiquitination (Figure 4H, Figure S7F). To define the residues relevant to the degradation activity, all NPM3 lysine residues were mutated to arginine (Figure 4I). Only the K102R mutation preserved NPM3 protein levels, and in denaturing IP, K102R abolished DTX3‐enhanced NPM3 ubiquitination (Figure 4J,K). DTX3 knockdown had the weakest effect on the ubiquitination of Lys102 (Figure S7G). Overall, DTX3 drives K27‐linked polyubiquitination at Lys102 of NPM3, promoting its degradation.

### 2.5 NPM3 Binds to RUVBL1 and Colocalizes with It in the Nucleus

MS identified NPM3‐interacting proteins (Figure 5A, Table S3). Among the top 10 proteins, RUVBL1, an AAA+ ATPase, was closely associated with HRR (Figure S8A). Exogenous and endogenous Co‐IP confirmed the interaction between NPM3 and RUVBL1, and this interaction remained unchanged over 24 h after irradiation (Figure 5B,C, Figure S8B). A GST pull‐down assay further demonstrated a direct NPM3–RUVBL1 interaction (Figure S8C). NPM3 and RUVBL1 colocalized in the nucleus after irradiation (Figure 5D, Figure S8D). However, western blotting showed that NPM3 did not affect RUVBL1 protein levels post‐irradiation (Figure 5E, Figure S8E). Consistently, DTX3 did not alter RUVBL1 expression post‐irradiation (Figure 5F,G); moreover, in DTX3‐overexpressing cells, NPM3 overexpression did not modify RUVBL1 levels with or without irradiation (Figure 5G). Overall, NPM3 directly binds to RUVBL1 without altering its expression, and the DTX3–NPM3 axis does not affect RUVBL1 expression.

**FIGURE 5:**
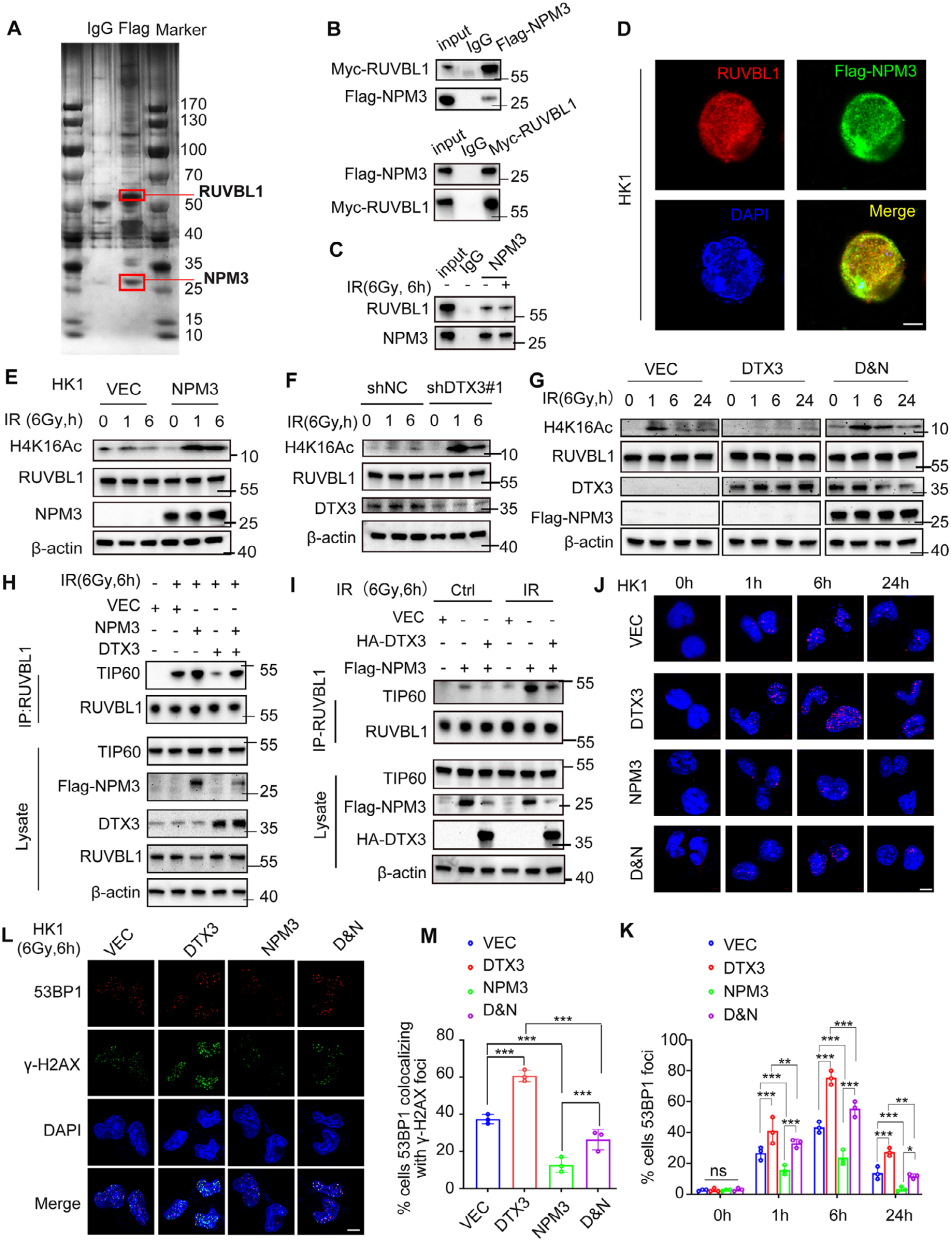
NPM3 binds to RUVBL1 and inhibits the formation of the RUVBL1‐TIP60 complex thereby hindering H4K16 acetylation. (A) Flag‐immunoprecipitated proteins from HK1 cells stably overexpressing Flag‐NPM3 were separated by SDS‐PAGE; proteins of interest are indicated by red lines. (B) NPM3–RUVBL1 exogenous interaction detected by Co‐IP in HK1 cells co‐expressing Flag‐NPM3 and Myc‐RUVBL1. (C) The endogenous association of NPM3 and RUVBL1 was detected by Co‐IP in HK1 cells with or without irradiation treatment. (D) Immunofluorescence staining showed nuclear colocalization of exogenous Flag‐NPM3 (green) and endogenous RUVBL1 (red) at 6 h following exposure to 6 Gy in HK1 cells. Scale bars: 5 µm. (E) The HK1 cells that overexpressed NPM3 or not were irradiated with 6 Gy and analyzed using immunoblot with H4K16Ac, RUVBL1, and NPM3 antibodies. (F) The HK1 cells in which DTX3 was knocked down or not were irradiated with 6 Gy and analyzed using immunoblot with H4K16Ac, RUVBL1, and NPM3 antibodies. (G) Involvement of NPM3 in DTX3‐mediated H4K16 acetylation. (H) The indicated HK1 cells were subjected to 6 Gy irradiation or not, and a Co‐IP assay was used to investigate the effect of the DTX3‐NPM3 axis on the formation of the RUVBL1‐TIP60 complex. (I) HK1 cells were transfected with Flag‐*NPM3* plasmids in the presence or absence of the HA‐*DTX3* expression plasmid, and Co‐IP was used to detect the effect of the DTX3‐NPM3 axis on the formation of the RUVBL1‐TIP60 complex before and after irradiation. (J,K) HK1 cells transfected with the indicated plasmids were irradiated with 6 Gy, then collected and incubated with the anti‐53BP1 antibody. Above: representative fluorescence images (scale bars, 10 µm; red, 53BP1; blue, DAPI) (J). Under: the percentage of positive cells with 53BP1 foci (>10) (K). Each datum point contains at least 20 cells. Data are presented as the mean ± SD (n = 3 independent experiments; two‐way ANOVA; ^*^ *p* < 0.05, ^**^ *p* < 0.01, ^***^ *p* < 0.001). (L,M). The indicated cells were treated with 6 Gy irradiation and co‐stained for γ‐H2AX and 53BP1 foci at the indicated time points (L). Quantification of 53BP1 colocalizing with γ‐H2AX foci (>10) (M) is shown (scale bars, 10 µm; red, 53BP1; green, γ‐H2AX, blue, DAPI). Data are presented as the mean ± SD (n = 3 independent experiments; one‐way ANOVA; ^***^ *p* < 0.001). n = 3 independent experiments. Abbreviations: IR, irradiation; D&N, DTX3+NPM3. The unprocessed images of the blots are shown in Figure S23.

### 2.6 DTX3–NPM3 Axis Hinders H4K16 Acetylation by Inhibiting the Formation of the RUVBL1–TIP60 Complex

Histone H4K16 acetylation is required to initiate HRR. NPM3 overexpression increased H4K16 acetylation (H4K16Ac), and this effect persisted following DTX3 knockdown (Figure 5E,F). Conversely, NPM3 knockdown or DTX3 overexpression reduced the H4K16Ac levels (Figure S8E, Figure 5G). Moreover, NPM3 overexpression reversed the DTX3‐induced decrease in H4K16Ac levels following irradiation (Figure 5G). Collectively, the DTX3–NPM3 axis inhibits HRR by suppressing H4K16 acetylation.

RUVBL1 often forms a complex with acetyltransferase TIP60 to acetylate histone H4K16, thereby promoting HRR‐mediated DSB repair [23]. Therefore, we investigated whether the DTX3–NPM3 axis regulates H4K16 acetylation by modulating the formation of the RUVBL1–TIP60 complex. Our results showed that NPM3 promoted the interaction between RUVBL1 and TIP60, whereas DTX3 inhibited this interaction (Figure 5H). Moreover, DTX3 inhibited the NPM3‐induced increase in RUVBL1–TIP60 complex formation (Figure 5H), and irradiation further augmented this effect (Figure 5I). This may be related to the enhanced HRR after irradiation. Overall, these findings indicated that the DTX3–NPM3 axis inhibited the formation of the RUVBL1–TIP60 complex, thereby suppressing H4K16 acetylation.

Based on the mechanism by which RUVBL1–TIP60 complex‐dependent H4K16 acetylation impairs recruitment of 53BP1 to DSB sites and facilitates HRR [24, 25], we propose that the DTX3–NPM3 axis governs the dynamic localization of 53BP1 at DSB lesions, thereby modulating HRR‐dependent DSB repair. To test this hypothesis, we assessed the effect of the DTX3–NPM3 axis on 53BP1 foci formation and disappearance by performing immunofluorescence analysis after irradiation. Our results showed that compared with the control cells, DTX3‐overexpressing cells exhibited more 53BP1 foci after irradiation, whereas NPM3‐overexpressing cells exhibited significantly fewer 53BP1 foci. Moreover, DTX3 overexpression counteracted the reduction in 53BP1 foci induced by NPM3 overexpression (Figure 5J,K, Figure S8F). These findings suggested that the DTX3–NPM3 axis inhibits 53BP1 foci formation in the nucleus. Additionally, in irradiated cells, 53BP1 colocalized with γ‐H2AX at multiple foci (Figure S8G), consistent with previous reports [23, 26, 27]. Further analysis of 53BP1 and γ‐H2AX colocalized foci revealed that DTX3 overexpression promoted 53BP1 recruitment to DSB sites, whereas NPM3 overexpression attenuated this effect (*p* < 0.001; one‐way ANOVA; Figure 5L,M). Collectively, these results indicated that the DTX3–NPM3 axis inhibits the assembly of the RUVBL1–TIP60 complex, reduces H4K16 acetylation, promotes 53BP1 binding at DSB sites, and ultimately diminishes HRR efficiency.

### 2.7 Elevated NPM3 Reverses the Radiosensitizing Effect of DTX3 In Vitro and In Vivo by Promoting the HRR Pathway

DTX3 reverses tumor radioresistance, and NPM3 is a downstream effector of DTX3. GSEA suggested that high NPM3 expression was correlated with pathways related to radiation response and DDR (Figure S9A). Therefore, we hypothesized that NPM3 promotes radioresistance in tumor cells. To test this hypothesis, we knocked down or overexpressed NPM3 in HK1 and HeLa cells (Figure S9B–E). NPM3 knockdown reduced radioresistance (*p* < 0.001; Figure 6A, Figure S10A), inhibited irradiation‐induced proliferation (*p* = 0.006, HK1; *p* = 0.030, HeLa; Figure 6B, Figure S10B), and promoted apoptosis (*p* < 0.001; Figure 6C, Figure S10C). In contrast, NPM3 overexpression increased radioresistance (*p* < 0.001; Figure S11A–D), promoted cell proliferation (*p* = 0.019, HK1; *p* = 0.033, HeLa; Figure S11B–E), and reduced apoptosis (*p* < 0.001; Figure S11C–F). Furthermore, NPM3 knockdown increased the comet tail moment after irradiation (*p* < 0.001; Figure 6D, Figure S12A), and NPM3‐knockdown cells exhibited high γ‐H2AX levels and more γ‐H2AX–positive foci (*p* < 0.001; Figure 6E,F, Figure S12B,C). HRR efficiency was significantly reduced by NPM3 knockdown, whereas NHEJ activity was unaffected (*p* < 0.001; Figure 6G,H, Figure S12D,E). The opposite effects were observed following NPM3 overexpression (Figure S13A–J). Collectively, these findings indicated that NPM3 promotes HRR and contributes to tumor cell radioresistance.

**FIGURE 6:**
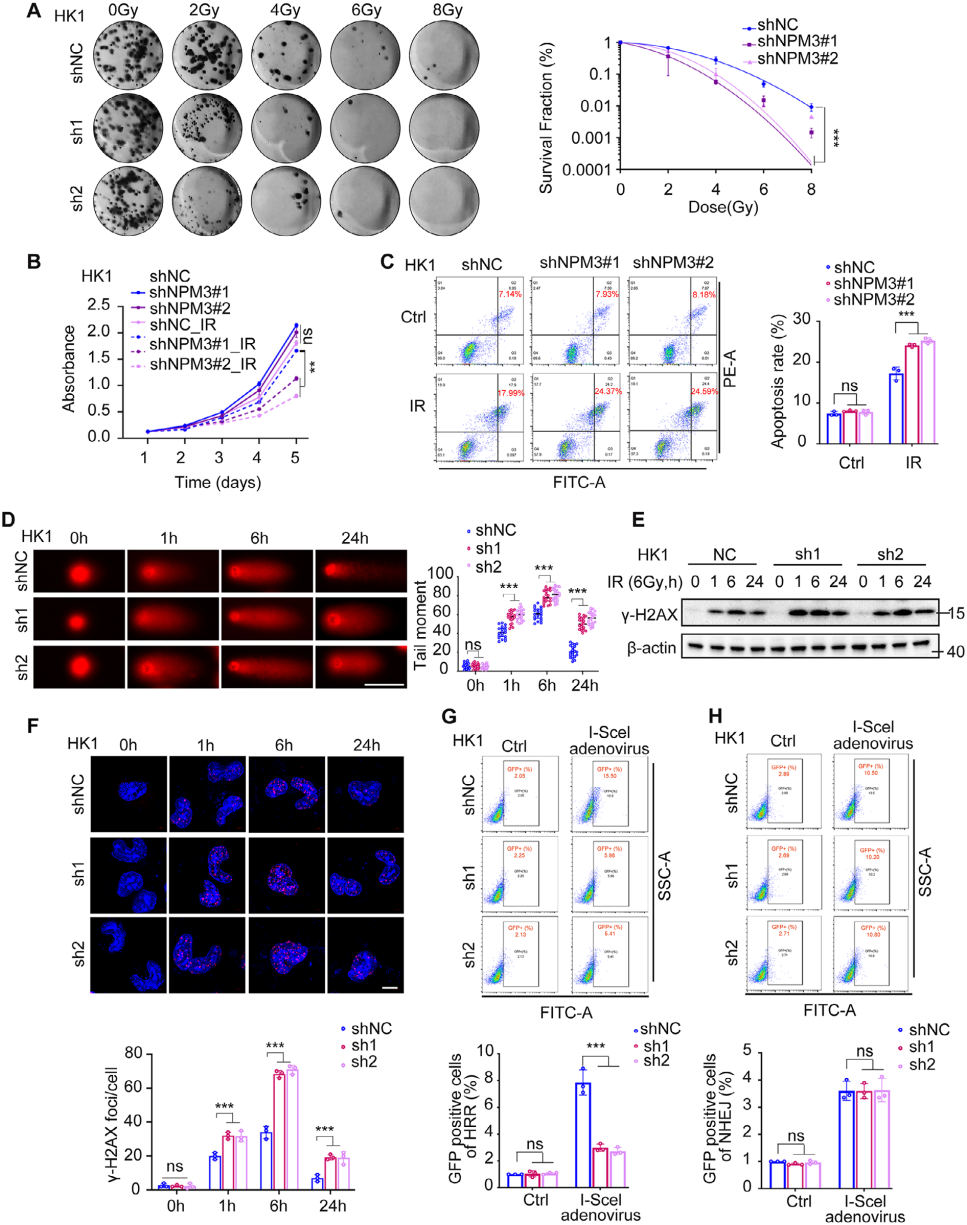
NPM3 knockdown inhibits radioresistance in tumors by impairing the HRR pathway in vitro. (A,B) HK1 cells with or without NPM3 knockdown were exposed to the indicated irradiation for clonogenic survival assays (A) and to 6 Gy irradiation for CCK‐8 viability assays (B). Data are presented as the mean ± SD (n = 3; extra sum‐of‐squares F test was used for A, two‐way ANOVA was used for B; ^**^ *p* < 0.01, ^***^ *p* < 0.001). (C) Flow cytometric analysis of apoptosis in HK1 cells with NPM3 knockdown at 24 h after exposure to either no irradiation or 6 Gy irradiation. Data are presented as the mean ± SD (n = 3; one‐way ANOVA; ***p < 0.001). (D). HK1 cells with or without NPM3 knockdown were collected at the indicated time points after 6 Gy irradiation for the neutral comet assay. Tail moment was analyzed with CaspLab‐Comet Assay software, scale bar: 50 µm. Data are presented as the mean ± SD (n = 20; two‐way ANOVA; ^***^p < 0.001). The experiments were repeated three times. (E). The expression of γ‐H2AX in NPM3 knockdown or not cells after irradiation was analyzed by western blotting. (F). Representative fluorescence images and quantification of γ‐H2AX foci in HK1 cells with or without NPM3 knockdown. Above: representative fluorescence images (scale bars, 10 µm; red, γ‐H2AX; blue, DAPI). Under: percentage of positive cells with γ‐H2AX foci (> 10). Each datum point contains at least 20 cells. Data are presented as the mean ± SD (n = 3 independent experiments; two‐way ANOVA; ^***^ *p* < 0.001). (G,H). Characterization of DSB repair pathways using HRR (G) and NHEJ (H) reporter assays. The proportion of GFP^+^ cells was measured by flow cytometry and the efficiency was normalized to the control cells. Data are presented as the mean ± SD (n = 3 independent experiments; one‐way ANOVA; ns, not significant; ^***^ *p* < 0.001). n = 3 independent experiments. Abbreviations: IR, irradiation. The unprocessed images of the blots are shown in Figure S23.

To verify the targeting specificity of sh*NPM3*, rescue experiments were conducted (Figure S14A). Re‐expression of NPM3 in sh*NPM3* HK1 or HeLa cells reversed the phenotypes induced by NPM3 knockdown, including reduced clonogenic survival (Figure S14B,C), increased apoptosis (Figure S15), and impaired HRR efficiency (Figure S16).

To confirm the DTX3–NPM3 axis effect in vitro and in vivo following irradiation, we generated VEC, DTX3, and DTX3+NPM3 cells (Figure S17A,B). The suppressive effects of DTX3 overexpression on tumor cell survival and proliferation were almost completely reversed by NPM3 overexpression (Figure 7A,B, Figure S18A,B), and DTX3‐induced apoptosis was attenuated by NPM3 overexpression after irradiation (*p* < 0.001; Figures S18C and S19). In vivo, tumor weight and volume were significantly reduced in the irradiated DTX3 group compared with the VEC group, whereas these two parameters were significantly increased in the NPM3 group. Compared to the NPM3 group, the DTX3+NPM3 group showed significantly decreased tumor weight and volume (Figure 7C–E). These results indicated that the radioresistance effect caused by NPM3 overexpression was almost completely reversed by DTX3 overexpression, suggesting that the DTX3–NPM3 axis is involved in inhibiting the radioresistance of tumors.

**FIGURE 7:**
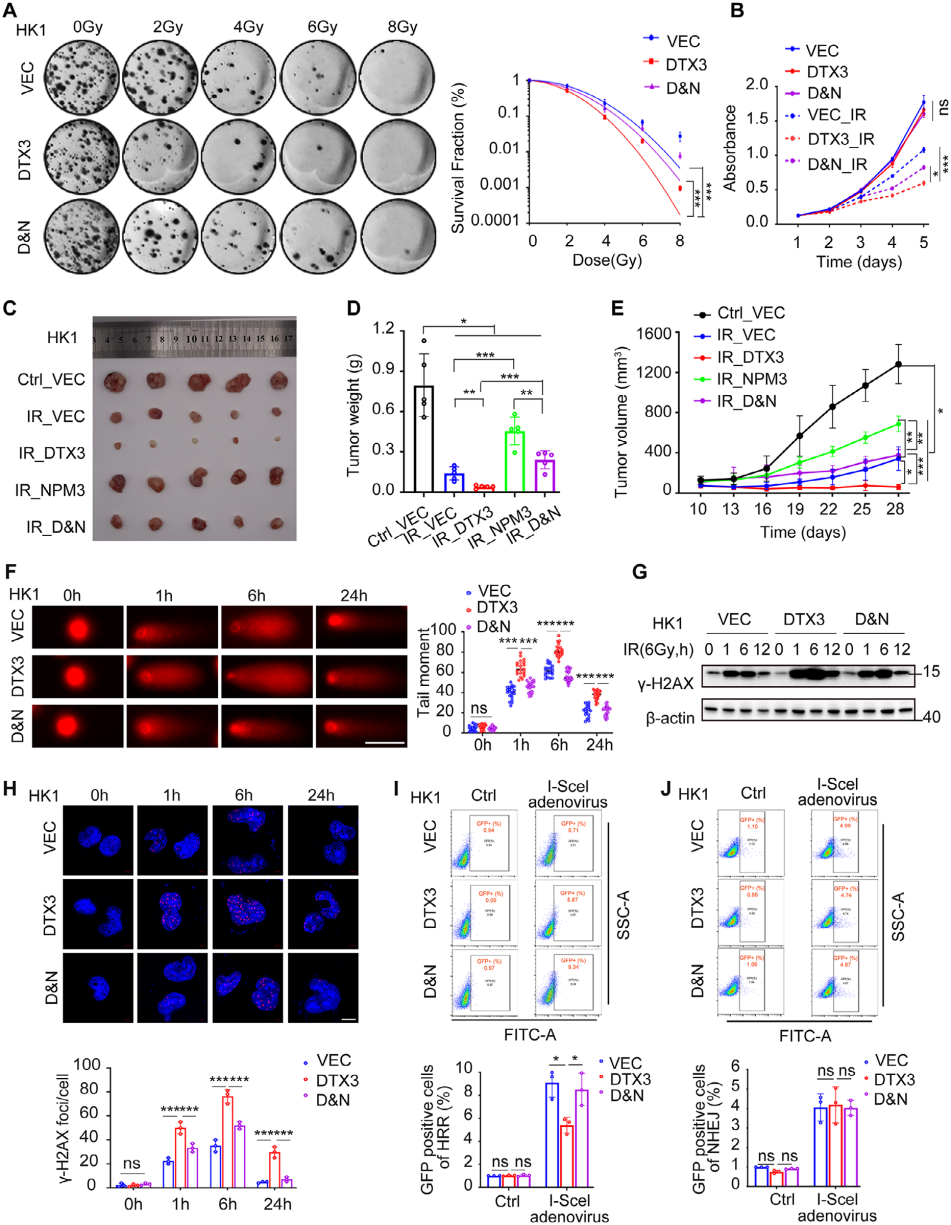
Elevated NPM3 reverses the radiosensitizing effect of DTX3 in vitro and in vivo by promoting the HRR pathway. (A,B). The indicated HK1 cells were exposed to indicated irradiation for clonogenic survival assays (A) and to 6 Gy for CCK‐8 viability assays (B) Data are presented as the mean ± SD (n = 3; extra sum‐of‐squares F test was used for A, two‐way ANOVA was used for B; ^*^ *p* < 0.05, ^***^ *p* < 0.001). (C,D,E) Subcutaneous xenografts were generated by implanting HK1 cells stably transfected into female BALB/c nude mice, followed by either irradiation with 8 Gy or not. Representative images (C), tumor weight (D), and tumor volume (E) are shown for each group (n = 5; unpaired t‐test for D, two‐way ANOVA for E; ^*^ *p* < 0.05, ^**^ *p* < 0.01, ^***^ *p* < 0.001). (F) Representative comet images and quantitative analysis of tail moments at the indicated time points after 6 Gy irradiation, scale bar: 50 µm. Data are presented as the mean ± SD (n = 20; two‐way ANOVA; ^***^ *p* < 0.001). (G) The expression of γ‐H2AX in indicated cells after irradiation was analyzed by western blotting. (H) Representative fluorescence images and quantification of γ‐H2AX foci immunostaining in indicated HK1 cells. Above: representative fluorescence images (scale bars, 10 µm; red, γ‐H2AX; blue, DAPI). Under: the percentage of positive cells with γ‐H2AX foci (> 10). Each datum point contains at least 20 cells. Data are presented as the mean ± SD (n = 3 independent experiments; two‐way ANOVA; ^***^ *p* < 0.001). (I,J) Characterization of DSB repair pathways by HRR (I) and NHEJ (J) reporter assays. The proportion of GFP^+^ cells was measured by flow cytometry and the efficiency was normalized to the control cells. Data are presented as the mean ± SD (n = 3 independent experiments; one‐way ANOVA; ns, not significant; ^*^ *p* < 0.05). n = 3 independent experiments. Abbreviations: IR, irradiation; D&N, DTX3+NPM3. The unprocessed images of the blots are shown in Figure S23.

To determine whether DTX3 suppresses HRR by degrading NPM3 and thereby reverses radioresistance, we performed a comet assay. NPM3 overexpression after irradiation reversed the increase in tail moment induced by DTX3 overexpression (*p* < 0.001; Figure 7F, Figure S20A). NPM3 overexpression also reversed the DTX3‐induced increase in γ‐H2AX levels and γ‐H2AX–positive foci (*p* < 0.001; Figure 7G,H, Figure S20B,C). In the HRR reporter assay, DTX3+NPM3 cells exhibited a higher proportion of GFP^+^ cells than DTX3 cells, whereas the GFP^+^ proportion in the NHEJ assay remained unchanged, indicating that NPM3 overexpression restored HRR activity without affecting NHEJ (*p* = 0.033, HK1; *p* < 0.001, HeLa; Figure 7I,J, Figure S20D,E). These results demonstrated that DTX3 targets NPM3 to inhibit HRR, thereby decreasing tumor radioresistance.

### 2.8 Low Expression of DTX3 Correlates with Unfavorable RT Prognosis

To determine the clinical significance of DTX3 expression in RT, we performed immunohistochemistry (IHC) staining of NPC tissues from 193 patients who had undergone RT. Based on the staining intensity, patients’ samples were categorized into a high DTX3 expression group (n = 96%, 49.7%) and a low DTX3 expression group (n = 97%, 50.3%) (Figure 8A). By integrating the IHC results with clinical data, we found that locoregional recurrence was related to weak DTX3 staining in NPC samples (Figure 8B). As shown in Figure 8B, the 15‐year locoregional recurrence rate was 0.031 (3/96) among patients with high DTX3 expression and 0.134 (13/97) among those with low DTX3 expression. Therefore, compared with patients with low DTX3 expression, those with high DTX3 expression had a relative risk (RR) of 0.231 (0.031/0.134) for locoregional recurrence, indicating that the risk of locoregional recurrence within 15 years was approximately 23.1% of that in patients with low DTX3 expression. Kaplan–Meier analysis revealed that low DTX3 expression was correlated with worse locoregional recurrence‐free survival (LRFS), disease‐free survival (DFS), and overall survival (OS) (Figure 8C–E). Multivariate analysis revealed that high DTX3 expression was independently associated with longer LRFS, DFS, and OS (Figure S21A–C). These results revealed that low expression of DTX3 predicted poor prognosis and was associated with locoregional recurrence in patients with NPC.

**FIGURE 8:**
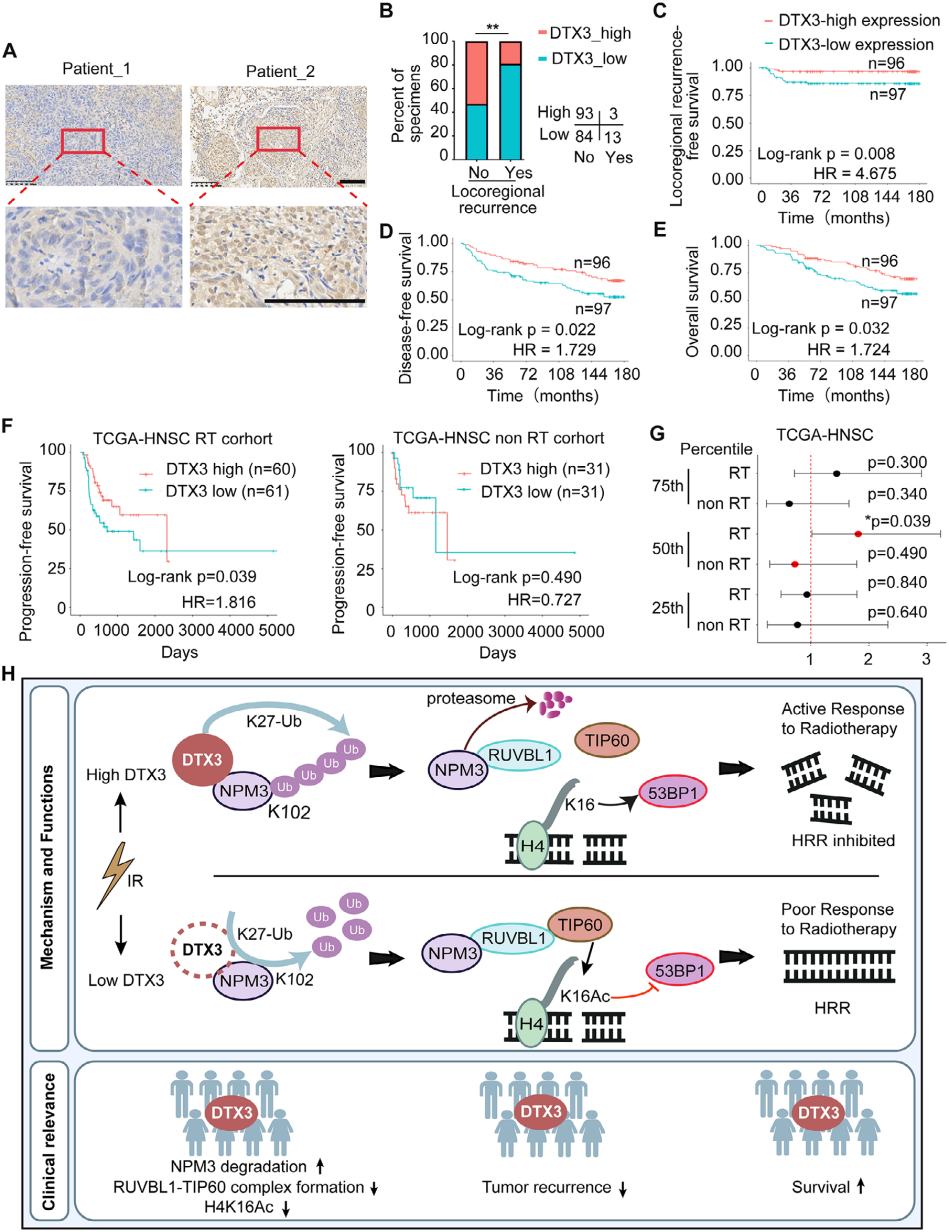
Low expression of DTX3 correlates with unfavorable radiotherapy (RT) prognosis. (A) Representative images of immunohistochemistry staining for anti‐DTX3 in 193 patients with NPC. Scale bars: 100 µm. (B) The correlation between locoregional recurrence status and the expression level of DTX3 was analyzed by the two‐tailed χ2 test. (C,D,E) Kaplan–Meier analysis of locoregional recurrence‐free survival (LRFS, **C**), disease‐free survival (DFS, **D**) and overall survival (OS, **E**) according to DTX3 expression levels using the log‐rank test. (F) Kaplan–Meier analysis of progression‐free survival (PFS) according to DTX3 expression in the TCGA‐HNSC dataset, stratified by RT status. The median was used as the cut‐off point. Data were presented as forest plots based on Hazard Ratio (HR) and 95% confidence intervals. The red dashed line indicated HR = 1. p‐values were determined using the log‐rank test with *p* < 0.05 considered statistically significant. (G) Kaplan–Meier analysis of PFS for the 25th, 50th, 75th percentiles of DTX3 expression in the TCGA‐HNSC dataset. (H) Proposed working model of DTX3. Low DTX3 expression is associated with poor prognosis and increased tumor recurrence. Mechanistically, DTX3 binds to NPM3 and promotes its ubiquitin‐dependent degradation, which disrupts the formation of the RUVBL1‐TIP60 complex. This disruption leads to reduced histone H4K16 acetylation and consequently diminished homologous recombination efficiency. Abbreviations: HNSC, head and neck squamous cell carcinoma.

Survival analysis of TCGA data showed that low DTX3 expression was associated with poor OS and progression‐free survival (PFS) in patients with low‐grade glioma and diffuse glioma (Figure S21D,E). When stratified by RT status, low DTX3 expression was associated with poorer PFS among patients with HNSC who received RT (Figure 8F,G). In bladder urothelial carcinoma and uterine carcinosarcoma, RT‐treated patients with low DTX3 expression had poorer OS than those with high DTX3 expression. However, no similar association was observed among non‐RT patients (Figure S22A–D). In lung adenocarcinoma (LUAD), low DTX3 expression was more strongly associated with poor OS and PFS in RT‐treated patients than among non‐RT patients (Figure S22E–H). In summary, low DTX3 expression is associated with unfavorable prognosis and a poor response to RT.

## 3 Discussion and Conclusions

Our study identified regulators of HRR suppression following RT. DTX3 was identified as a key molecule suppressing HRR and reversing tumor radioresistance via ubiquitination‐mediated regulation of the NPM3–RUVBL1 axis. Mechanistically, DTX3 binds NPM3 and facilitates the ubiquitin‐dependent degradation of NPM3, thereby inhibiting the formation of the RUVBL1–TIP60 complex, reducing histone H4K16 acetylation, and consequently decreasing HRR efficiency. Clinically, low DTX3 expression is associated with poor prognosis and correlates with locoregional recurrence after RT. Our findings provided mechanistic support for DTX3, an E3 ubiquitin ligase, as a modulator of tumor radiosensitivity and identified a potential target to overcome tumor radioresistance (Figure 8H).

DTX3 is a member of the DTX family, with a C‐terminal RING‐type E3 ligase domain, a motif rich in prolines, and an N‐terminal WWE‐rich region. Notably, the structure of DTX3 contains a special truncated NTD that lacks homology with other DTX proteins [28]. DTX3 is primarily expressed in the nucleus and plays a dual role in the invasion, metastasis, and proliferation of various tumors. As a tumor suppressor, DTX3 ubiquitinates NOTCH2 to inhibit the proliferation and migration of esophageal cancer cells [29]. In triple‐negative breast cancer, the DTX3‐mediated ubiquitination of NOTCH4 is diminished by DCAF13, resulting in enhanced tumor invasion and migration [30]. In papillary thyroid carcinoma, DTX3 promotes the ubiquitination of XRCC5 to suppress epithelial–mesenchymal transition [31]. In bladder cancer, DTX3 suppresses invasion and metastasis by degrading NICD, thereby inhibiting Notch signaling [32]. Conversely, Wang et al. reported that DTX3, as an oncogenic factor, promotes ovarian cancer growth by stabilizing mutant p53 through disruption of the MDM2–mutant p53 interaction [33]. These diverse functions highlight the multifaceted roles of DTX3, likely arising from substrate selectivity in different cellular contexts. In this study, we identified two notable characteristics of DTX3: first, it is strongly associated with RT response and tumor radioresistance; second, it inhibits RUVBL1–TIP60 complex formation by targeting and promoting the K27‐linked ubiquitination‐mediated degradation of NPM3, thereby preventing DDR signal transduction. Our findings contributed to a more comprehensive understanding of the mechanisms by which DTX3 inhibits tumor radioresistance and further illustrated the complexity of ubiquitination regulatory networks in tumor progression.

Such opposing tumor‐suppressive and oncogenic activities indicate that DTX3 functions in a context‐dependent manner, influenced by intrinsic cellular characteristics and extrinsic environmental signals. Our findings suggest that DNA damage stress may be an important factor influencing DTX3 function. Under irradiation, DTX3 preferentially interacts with NPM3, promoting its degradation and thereby inhibiting RUVBL1‐TIP60 complex formation. This process reduces H4K16 acetylation and impairs HRR. NPM3 represents a newly identified substrate of DTX3, adding to the panel of known DTX3 substrates including NOTCH family proteins, XRCC5 and mutant p53. Here, several factors may influence the functional consequences of DTX3 activity, including (1) NPM3 expression levels in tumor cells, (2) p53 status, and (3) the availability of specific substrates within the tumor microenvironment. In tumors with high NPM3 expression and intact p53 signaling, DTX3‐mediated NPM3 degradation may contribute to tumor suppression and enhance radiosensitivity. In contrast, in tumors with low NPM3 expression, mutant p53 mutations, or other predominant DTX3 substrates, such as NOTCH family proteins, XRCC5, or mutant p53, DTX3 may exert different biological effects through alternative substrates. Further studies are needed to determine how these factors interact to shape the biological functions of DTX3 and whether this context dependence affects its potential as a therapeutic target across different tumor types.

Ubiquitination regulates multiple cellular processes. Although canonical K48‐ and K63‐linked ubiquitination are well characterized, noncanonical ubiquitination remains poorly understood. In recent years, K27‐linked ubiquitination has attracted broad attention because of its diverse biological functions across various processes, including protein degradation, selective autophagy, regulation of kinase activity, subcellular localization of proteins, protein–protein interactions, and signal transduction [34]. Various E3 ligases promote substrate degradation by mediating K27‐linked ubiquitination. For example, DTX2 degrades TfR1 via K27‐linked ubiquitination to prevent sepsis‐induced myocardial injury [35]. The E3 ubiquitin ligase MARCH2 promotes K27‐linked ubiquitination and degradation of TNF‐R1 at lysine 340 [36]. The E3 ligase MYLIP targets HIF‐1α and catalyzes K27‐linked polyubiquitination, subsequently inducing proteasomal degradation of HIF‐1α [37]. We revealed that the E3 ligase DTX3 mediates NPM3 degradation through K27‐linked ubiquitination and participates in DSB repair signaling, providing mechanistic insights into DDR induction by irradiation.

The NPM family includes NPM1/2/3 and participates in chromatin remodeling, genomic stability, ribosome biogenesis, DNA replication, and transcription, with significant implications for tumorigenesis [38]. NPM3 is a predominantly nuclear, nucleic acid–binding protein associated with chromatin [39]. NPM3 mediates immune escape in gastric cancer by promoting the transcriptional activation of NPM1 [40]; NPM3 also promotes the proliferation and migration of human LUAD cells in vitro [41]. Gu et al. found that NPM3 is overexpressed in prostate cancer and may serve as a marker of disease invasiveness [42]. However, to date, there are no reports on the function and mechanism of NPM3 in tumor radiosensitivity. Here, NPM3 was identified as a novel ubiquitin‐substrate of DTX3, which is associated with enhanced radioresistance. DTX3 mediates ubiquitination and proteasomal degradation of NPM3, inhibiting HRR and overcoming tumor radioresistance. This finding expands our understanding of the regulatory mechanisms by which NPM3 modulates tumor radioresistance.

RUVBL1 is an AAA+ ATPase that participates in diverse chromatin remodeling functions [43]. It plays crucial roles in both NHEJ and HRR. Ubiquitinated RUVBL1 can interact with β‐catenin, thereby enhancing NHEJ repair in breast cancer cells [4]. Additionally, RUVBL1 is typically associated with RuvB‐like protein 2 and TIP60, localizes to the nucleus, and promotes the formation of H4K16Ac, which suppresses the binding of 53BP1 to DSB sites and promotes HRR [23]. Our findings indicated that RUVBL1 directly interacts with NPM3, and the DTX3–NPM3 axis inhibits the formation of the RUVBL1–TIP60 complex, thereby reducing H4K16 acetylation and promoting the recruitment of 53BP1 to DSB, which impedes HRR. Overall, we delineated the regulatory effects of the DTX3–NPM3 axis on RUVBL1–TIP60 complex formation and its potential mechanism in modulating HRR.

Although this study elucidated the role of the DTX3–NPM3–RUVBL1 axis in reversing tumor radioresistance, some limitations should be acknowledged and addressed in future studies. First, clinical evidence is largely derived from a single‐center cohort and public datasets; validation in multicenter cohorts spanning diverse tumor types is needed to confirm the universality and specificity of the axis expression patterns and radiosensitivity relationships. Second, our experimental systems predominantly rely on in vitro cell assays and subcutaneous xenograft models, which may not fully recapitulate the complexity of human tumors in vivo; future studies should incorporate orthotopic tumor models and patient‐derived xenografts to better simulate RT responses in a human physiological context. Third, although we demonstrated that DTX3 reverses radioresistance by degrading NPM3 and enhances radiosensitivity, the clinical translational potential and feasibility remain to be systematically evaluated. Future efforts should focus on specificity‐oriented interventions that promote DTX3 expression or augment its E3 ligase activity and assess therapeutic efficacy, safety, and the potential for resistance development.

To conclude, our data suggested an important role of the DTX3–NPM3–RUVBL1 axis in the regulation of tumor DDR signaling and radiosensitivity (Figure 8H). Low DTX3 expression is associated with poor response to RT, suggesting that DTX3 may serve as a biomarker and therapeutic target for inhibiting radioresistance in cancer, warranting further investigation as a potential combination therapy with RT.

## 4 Materials and Methods

### 4.1 Patients and Tumor Specimens

Paraffin‐Embedded tissue specimens were collected from 193 patients diagnosed with locally advanced NPC between October 2011 and August 2022. All biopsy samples were obtained before patients received any antitumor therapy. Clinicopathological data of this cohort are summarized in Tables S4 and S5. The study was approved by the Institutional Ethical Review Board of Sun Yat‐sen University Cancer Center (Approval No. B2026‐128‐01), with informed consent exempted.

### 4.2 Cell Culture

The human NPC cell line HK1 (a gift from M.S. Zeng, Sun Yat‐sen University Cancer Center, Guangzhou, China) and cervical carcinoma cell line HeLa were cultured in RPMI‐1640 (Sigma‐Aldrich, USA) supplemented with 10% fetal bovine serum (FBS; Excell Bio, China). HEK293T cells were maintained in DMEM (Corning, USA) supplemented with 10% FBS. All cell lines were incubated at 37°C in a humidified incubator supplied with 5% CO_2_, and routine mycoplasma screening was performed to exclude contamination.

### 4.3 Plasmid and Lentivirus

The following plasmids were purchased from Kidan Bioscience (China): pCDH‐CMV‐*DTX3*‐HA‐EF1‐Puro, plko‐u6‐shRNA#1/2(*DTX3*, human)‐G418, plv‐cmv‐mcs‐ef1‐puro‐*Ub(K6)*‐HA, plv‐cmv‐mcs‐ef1‐puro‐*Ub(K11)*‐HA, plv‐cmv‐mcs‐ef1‐puro‐*Ub(K27)*‐HA, plv‐cmv‐mcs‐ef1‐puro‐*Ub(K29)*‐HA, plv‐cmv‐mcs‐ef1‐puro‐*Ub(K33)*‐HA, plv‐cmv‐mcs‐ef1‐puro‐*Ub(K48)*‐HA, plv‐cmv‐mcs‐ef1‐puro‐*Ub(K63)*‐HA, pCDH‐CMV‐MCS‐EF1‐NEO‐3Flag‐*NPM3*, pLV‐u6‐shRNA#1/2(*NPM3*, human)‐EF1‐Puro, pCMV‐*DTX3‐ΔRING* (167‐208aa)‐HA, pCMV‐*NPM3(K53R)*‐3×Flag, pCMV‐*NPM3(K76R*)‐3×Flag, pCMV‐*NPM3(K102R)*‐3×Flag, pCMV‐*NPM3(K125R)*‐3×Flag, pCMV‐*NPM3(K172R)*‐3×Flag, pCMV‐*NPM3(K173R)*‐3×Flag, pCDH‐CMV‐h‐*TIP60*‐HA‐EF1‐Puro, and pCDH‐CMV‐h‐*RUVBL1*‐Myc‐EF1‐Puro. Mutant plasmids of HA‐DTX3 and Flag‐NPM3 were also obtained from Kidan Bioscience. *shRNA* sequences are listed in Table S6.

Plasmids containing the gene described above were transfected into tumor cells using Lipofectamine 3000 (Invitrogen, USA). The plko‐u6‐shRNA#1/2(*DTX3*, human)‐G418 plasmids were transiently transfected into the stable DTX3‐overexpressing HK1 and HeLa cells because DTX3 expression in these cells was relatively low. For stable transduction into cancer cells, HEK293T cells were co‐transfected with the lentivirus packaging plasmids pMD2.G and pSPAX2 using Lipofectamine 2000, and the viral supernatant was collected 48 h post‐transfection. Cancer cells were infected with the produced viral supernatant and subjected to continuous selection with puromycin (1 g/mL, Sigma‒Aldrich, USA) or G418 (400 µg/mL, Beyotime Biotechnology, China) for 7–10 days. The resulting cell lines were subjected to reverse transcription quantitative PCR (RT‐qPCR) or western blot analyses.

### 4.4 Clonogenic Assay

Cells were seeded in six‐well plates at 200–10,000 cells/well and irradiated with 0–8 Gy after ensuring cell adhesion. After colony growth (10–14 days), the wells were washed with phosphate‐buffered saline (PBS), fixed with 4% paraformaldehyde, and stained with crystal violet. Colonies containing at least 50 cells were counted. The surviving fraction was modeled using the linear‐quadratic function surviving fraction = exp (−*αD*− *βD* ^2^) to fit the cell survival curves.

### 4.5 RNA Extraction and RT‐qPCR

RNA was extracted using the TRIzol reagent (Invitrogen, USA) according to the manufacturer's protocol. Complementary DNA was synthesized using a reverse transcription kit (TaKaRa, Japan) following the manufacturer's instructions. RT‐qPCR was performed in a 384‐well plate using the SYBR Green PCR Kit (TaKaRa, Japan) and an ABI Prism 7900HT Sequence Detector (Applied Biosystems, USA). Relative gene expression was calculated using the 2^−ΔΔCT^ method, with GAPDH as the endogenous control. The primer sequences are listed in Table S6.

### 4.6 Western Blotting

Proteins were extracted from the cells using on‐ice sonication after cell lysis. The extracted proteins were separated via SDS‐PAGE and transferred onto PVDF membranes (Millipore, USA). Membranes were blocked with 5% (w/v) bovine serum albumin (BSA; Sigma–Aldrich, USA) and incubated with primary antibodies overnight at 4°C. After washing, horseradish peroxidase‐conjugated secondary antibodies were applied, and the target proteins were detected using an enhanced chemiluminescence system (GE Healthcare). β‐Actin served as the loading control. The antibodies used in this study are listed in Table S7.

### 4.7 Cell Proliferation Assay

Cells were seeded at 2000 cells/well in 96‐well plates and either subjected to irradiation or left untreated. On days 1–5, 10 µL of Cell Counting Kit‐8 reagent (TargetMol, USA) was added to each well. Following a 2 h incubation at 37°C, absorbance was measured at 450 nm using a spectrophotometer to assess cell viability.

### 4.8 Flow Cytometry Analysis of Apoptosis

Apoptosis was assessed using an Annexin V‐FITC/Propidium Iodide (PI) Apoptosis Detection Kit (Yeasen Biotech, China). Cells were collected 24 h with or without 6 Gy irradiation, washed twice with PBS, and resuspended in 500 µL of binding buffer. Samples were stained with 5 µL of Annexin V‐FITC and 10 µL of PI and incubated in the dark. Flow cytometry was performed using a flow cytometer (Beckman Coulter, China), and the data were analyzed using CytExpert 2.2 software. Viable cells were defined as Annexin V^−^/PI^−^, early apoptotic as Annexin V^+^/PI^−^, and late apoptotic or dead as Annexin V^+^/PI^+^.

### 4.9 Comet Assay

Tumor cells harvested at sequential time points post irradiation were subjected to DNA damage analysis using the SCGE DNA Damage Detection Kit (KeyGen, China) following neutral electrophoresis (KeyGen, China). Comet images were acquired at 20 × magnification using an inverted fluorescence microscope (Olympus IX73, Japan). Tail moment values were quantified with CaspLab Comet Assay software, with 20 cells selected for measurement per sample.

### 4.10 NHEJ and HRR Efficiency Detection

According to the method we previously reported [10], stable tumor cell lines expressing GFP‐HRR and GFP‐NHEJ reporters were generated via transfection, followed by G418 selection (0.5 mg/mL; Beyotime) for 10 days, during which no GFP signal was observed. Cells were co‐transfected with the indicated *DTX3* plasmid or *shRNAs* and the pCMV‐NLS‐I‐SceI vector to induce DSBs. GFP expression was activated only if HRR or NHEJ repaired DSBs. The GFP‐positive cell frequency, assessed 48 h after transfection using flow cytometry (Beckman Coulter, China), served as a readout of HRR or NHEJ repair activity. The HRR‐ or NHEJ‐mediated repair efficiency was expressed as the fraction of GFP^+^ cells normalized to that of the control.

### 4.11 Co‐IP Assay and MS Analysis

Protein complexes were immunoprecipitated from cell lysates using 3 µg of antibody, with overnight incubation at 4°C in IP lysis/wash buffer supplied with the Pierce Classic Magnetic IP/Co‐IP Kit (Cat. No. 88804, Thermo Scientific, USA). This buffer contained 25 mm Tris, 150 mm NaCl, 1 mm EDTA, 1% NP‐40, and 5% glycerol (pH 7.4). Immune complexes were captured using the kit's Protein A/G magnetic beads provided with the kit and subsequently washed five or six times with 500 µL of IP lysis/wash buffer per wash. The precipitates were resolved via SDS‐PAGE and visualized using a Fast Silver Stain Kit (Beyotime, China). MS analysis was performed by Wininnovat Biotech (China) and ELEM Biotech (China). Proteins present in the Co‐IP were further validated using western blotting. Table S7 lists the antibodies used in this study.

### 4.12 GST Pull‐Down Assay

A total of 500 µL of bacterial lysate (250 µg) expressing either GST or GST‐NPM3 (MabStar, K10466) was bound to 40 µL of glutathione Sepharose 4B beads (GE Healthcare, 17‐0756‐01). The resulting complexes were incubated overnight with whole‐cell lysates from HK1 cells overexpressing HA‐DTX3 or Myc‐RUVBL1 at 4°C. Following incubation, the beads were washed three times with 500 µL of PBS (pH 7.4; 137 mm NaCl, 2.7 mm KCl, 10 mm Na_2_HPO_4_, and 1.8 mm KH_2_PO_4_) supplemented with a fresh protease inhibitor cocktail and phosphatase inhibitor cocktail for each wash. Bound proteins were eluted by heating at 95°C for 10 min. Samples were analyzed via western blotting using antibodies against GST, DTX3, and Myc. Table S7 lists the antibodies used in this study.

### 4.13 Confocal Microscopy

Fixed‐cell immunofluorescence was performed using cells seeded on Milli‐EZSlide 8‐well Glass Slides (Merck Millipore, USA). A methanol:acetone fixative (1:1, precooled to −20°C) was used. Following 24 h of transfection with the relevant plasmids, the cells were placed on a slide, and irradiation was applied 12 h after attachment. At the indicated time points, slides were washed with PBS three times and fixed in −20°C fixative overnight. Staining was performed on the following day. Cells were washed three times with Tris‐buffered saline (TBS), blocked with 5% (w/v) BSA for 30 min, and incubated with primary antibodies at 4°C overnight. After washing, the corresponding fluorescent secondary antibodies were applied for 60 min at room temperature, followed by counterstaining with DAPI (Sigma–Aldrich, USA). To assess colocalization in tumor cells, the protocol was repeated for the second primary antibody. Fluorescent images were acquired using a laser‐scanning confocal microscope (LSM900, Fast Airyscan, Zeiss, Germany). Table S7 lists the antibodies used for the immunofluorescence analyses.

### 4.14 MG132, CQ, and CHX Treatments

Cells were treated with either 10 µm MG132 or 50 µm CQ (Sigma‒Aldrich, USA) for 6 h. To suppress protein synthesis, CHX was applied at 100 µg/mL for 0, 1, 6, and 12 h.

### 4.15 Denature‐IP Assay

Denature‐IP assays were conducted under denaturing conditions. Denaturing IP experiments were performed as previously described [3]. Cells were lysed on ice with IP lysis buffer supplemented with a protease inhibitor cocktail (Roche, Switzerland) 24 h after transfection. Lysates were subjected to IP using the same protocol as for Co‐IP, followed by denaturation at 95°C after exposure to 1% (w/v) SDS for 10 min. Immune complexes were analyzed using western blotting.

### 4.16 Animal Experiments

Animal experiments were conducted at the animal facilities of BeiGene (Guangzhou) Innovation Technology Co., Ltd. Female BALB/c nude mice (5 weeks old; n = 25) were obtained from Gempharmatech‐GD/GDMLAC (Guangdong, China) and housed under barrier conditions at 18°C–22°C, 50%–60% relative humidity, with a 12 h light/dark cycle. Mice were randomly inoculated subcutaneously with 3 × 10^5^ HK1 cells in the right flank. Tumor volumes were measured every 3 days beginning on day 7 after injection. When the tumor diameter reached 5 mm, the mice received local irradiation at either 0 or 8 Gy. Tumor volume was calculated as volume = length × (width)^2 × 0.5. All procedures complied with the ethical principles of animal research and were approved by the Institutional Animal Care and Use Committee of BeiGene (Guangzhou) Innovation Technology Co., Ltd (Approval No. 2025017). Humane endpoints were applied to minimize pain and distress, and the maximum permissible tumor diameter was 20 mm, which was not exceeded in any group.

### 4.17 IHC

Following deparaffinization, antigen retrieval was performed using a basic EDTA buffer (pH 9.0; Panovue, China) and microwave heating. Sections were washed twice in TBS, incubated with hydrogen peroxide, and blocked with goat serum (Beyotime, China) prior to overnight incubation with primary antibodies at 4°C. The next day, sections were warmed, washed three times with TBS, and incubated with the appropriate rabbit or mouse secondary antibody for 30 min according to the Dako REAL EnVision Detection System protocol (Dako, Denmark). Detection was performed using DAB staining followed by hematoxylin counterstaining. Two pathologists independently scored all sections using the Immune Response Score framework. The staining intensity was graded as: 0 (no staining), 1 (weak), 2 (moderate), and 3 (strong). The proportion of positively stained tumor cells was scored as follows: 0 (< 6%), 1 (6%–25%), 2 (26%–50%), 3 (51%–75%), or 4 (> 75%). Histochemistry (H)‐scores, ranging from 0 to 12, were calculated as the product of the intensity and percentage scores (H‐score = intensity × percentage) [10]. DTX3 expression was dichotomized using the median as the cutoff: H‐scores ≤4 were classified as low expression, and those >4 as high expression (Figure 8A–E). Images were captured using a digital pathology scanner (KF‐PRO‐020, China). The antibodies used for IHC are listed in Table S7.

### 4.18 Statistical Analysis and Reproducibility

Statistical analyses were conducted with GraphPad Prism (version 9.0), SPSS 24.0, or R software. Unpaired or paired two‐tailed Student's t‐tests and χ^2^ tests were applied for comparisons between two groups, whereas one‐way or two‐way ANOVA was used for multigroup comparisons. Clonogenic survival fraction curves were assessed by the extra sum‐of‐squares F test. Survival curves were plotted using the Kaplan–Meier method. All experiments were independently performed in biological triplicate. Data are expressed as the mean ± standard deviation (SD), and a *p* < 0.05 was considered statistically significant.

## Author Contributions

J.H.X., C.J.X., Y.H.C., and H.C. contributed equally to this study. P.F. conceived, designed, supervised the study, and secured funding. Y.Q.L. collected the clinical samples, clinical information, and follow‐up data, conducted IHC, and assisted with pathological analysis. J.H.X., C.J.X. and P.F. performed all in vitro experiments. P.F., Y.H.C. and H.C. finished xenograft in vivo studies. S.H.L., W.L., Y.W.B. and R.M.H completed bioinformatic analyses. Y.H.C. and H.C. offered partial funding. J.H.X. and P.F. drafted the manuscript. P.F. and Y.Q.L. revised the paper. All authors read and approved the final paper.

## Funding

This work was supported by the Natural Science Foundation of Hunan Province (Grant number: 2026JJ81669) and the Health Research Project of Hunan Provincial Health Commission (Grant number: 20255494).

## Ethics Statement

The Study Was Approved by the Sun Yat‐Sen University Cancer Center's Institutional Ethical Review Board (B2026‐128‐01) with Informed Consent exempted. Animal experiments Were Approved By the Institutional Animal Care and Use Committee of BeiGene (Guangzhou) Innovation Technology Co., Ltd and complied with the relevant ethical guidelines (2025017). All research protocols adhered to the Declaration of Helsin.

## Conflicts of Interest

The authors declare no conflicts of interest.

## Supporting information




**Supporting File**: advs78573‐sup‐0001‐SuppMat.docx.

## Data Availability

The data that support the findings of this study are available from the corresponding author upon reasonable request.
