# Supplementary material for: DTX3 Suppresses the NPM3–RUVBL1 Axis to Inhibit Homologous Recombination Repair and Abrogate Tumor Radioresistance
Source: Adv Sci (Weinh). 2026 Oct 29:e78573. Online ahead of print. doi: 10.1002/advs.78573 (PMC13720273; doi:10.1002/advs.78573)
Supplement: Supplementary file 1 [file ADVS-9999-e78573-s001.docx]

Supporting Information

**DTX3 Suppresses the NPM3–RUVBL1 Axis to** **Inhibit Homologous Recombination Repair and Abrogate Tumor Radioresistance**

*Jia-hao Xiao, Chang-jun Xie, Yan-hua Chen, Hao Cheng, Shuang-hui Li, Wei Li, Ya-wen Bin, Rui-min He, Ying-qing Li^*^, Ping Feng^*^*

Supplementary figures

Figure S1. DTX3 reduces tumor radioresistance by inhibiting proliferation and promoting apoptosis.

Figure S2. **DTX3 impairs DNA double-strand break (DSB) repair by inhibiting the homologous recombination repair (HRR) pathway in HeLa cells.**

Figure S3. Re-expression of DTX3 reverses the DTX3 knockdown-mediated promotion of clonogenic formation after irradiation.

Figure S4. Re-expression of DTX3 reverses the inhibition of apoptosis caused by DTX3 knockdown.

Figure S5. **Re-expression of DTX3 restores the promotion of HRR repair induced by DTX3 knockdown in HK1 and HeLa cells.**

Figure S6. **DTX3 binds to NPM3.**

Figure S7. DTX3 ubiquitinates and degrades NPM3.

Figure S8. **NPM3 binds to RUVBL1 and inhibits 53BP1 binding at DSB sites.**

Figure S9. The GESA analysis on NPM3 and t**he expression efficiency of cancer cells transfected with the indicated plasmids.**

Figure S10. **NPM3 knockdown decreases radioresistance in HeLa cells in vitro.**

Figure S11. **NPM3 overexpression promotes radioresistance in HK1 and HeLa cells in vitro.**

Figure S12. **NPM3 knockdown impaires HRR in HeLa cells.**

Figure S13. **NPM3 overexpression promotes HRR in HK1 and HeLa cells.**

**Figure** S14. Re-expression of NPM3 reverses the NPM3 knockdown-mediated promotion of clonogenic formation after irradiation.

Figure S15. Re-expression of NPM3 reverses the inhibition of apoptosis caused by NPM3 knockdown.

Figure S16. **Re-expression of NPM3 restores the promotion of HRR repair induced by NPM3 knockdown in HK1 and HeLa cells**

**Figure** S17. **The VEC, *DTX3*, and *DTX3+NPM3 (D&N)* HK1 or HeLa cells were stably established.**

**Figure** S18. **Elevated NPM3 reverses the radiosensitizing effect of DTX3 in HeLa cells.**

**Figure** S19. **Flow cytometric analysis of apoptosis in the indicated HK1 cells at 24 h after exposure to either no IR or 6 Gy irradiation.**

**Figure** S20. **Elevated NPM3 reverses DTX3 mediated inhibition of HRR in HeLa cells.**

**Figure** S21. Lower expression of DTX3 is associated with poor prognosis.

**Figure** S22. **Low expression of DTX3 correlates with unfavorable radiotherapyprognosis.**

Figure S23. Unprocessed immunoblots for indicated Figures panels

Supplementary tables

Table S1. **List of E3 ligase library (n=769)**

Table S2. **List of protein candidates that interact with HA‑DTX3 identified by mass spectrometry.**

**Table S3**. **List of protein candidates that interact with Flag-NPM3 identified by mass spectrometry.**

**Table S4**. **Correlations between DTX3 expression levels and clinical features in patients with nasopharyngeal carcinoma.**

**Table S5**. **Clinicopathological data of 193 paraffin-embedded NPC tissues for immunohistochemistry staining.**

**Table S6.** **List of primers and shRNA sequences used in this study.**

**Table S7. List of antibodies used in this study.**

Figure S1. DTX3 reduces tumor radioresistance by inhibiting proliferation and promoting apoptosis. A, B. Western blotting and RT-qPCR analysis of HK1 cells with DTX3 overexpression **(A)** and knockdown **(B)**. **C, D**, Western blotting and RT-qPCR analysis of HeLa cells with DTX3 overexpression (**C**) and knockdown (**D**). **E, F**. Clonogenic assays and survival fraction curves after exposure to indicated IR (**E**) and Cell Counting Kit-8 (CCK-8) assays after exposure to 6 Gy (**F**) of HeLa cells with/without DTX3 overexpression. Data are presented as the mean ± SD (n=3; extra sum-of-squares F test was used for **E**, two-way ANOVA was used for **F**; *p <0.05, ***p < 0.001). **G, H.** Clonogenic assays and survival fraction curves after exposure to indicated IR (**G**) and CCK-8 assays after exposure to 6 Gy (**H**) in HeLa cells with/without DTX3 knockdown. Data are presented as the mean ± SD (n=3; extra sum-of-squares F test was used for **G**, two-way ANOVA was used for **H**; *p < 0.05, ***p < 0.001). **I, J**. Flow cytometric analysis of apoptosis in HeLa cells with DTX3 overexpression (**I**) or knockdown (**J**) at 24 h after exposure to either no IR or 6 Gy IR. Data are presented as the mean ± SD (n=3; unpaired t-test was used for **I**, one-way ANOVA was used for **J**; ***p < 0.001). **K, L**. Schematic for detection of NHEJ (**K**) and HRR (**L**) repair efficiency. n=3 independent experiments. Abbreviations: IR, irradiation. The unprocessed images of the blots are shown in Figure S23 (Supporting Information).

Figure S2. **DTX3 impairs DNA double-strand break (DSB) repair by inhibiting the homologous recombination repair (HRR) pathway in HeLa cells. A**. HeLa cells with/without DTX3 overexpression were collected at the indicated time after 6 Gy IR for the neutral comet assay. CaspLab-Comet Assay software was used to analyze the tail moment, scale bar: 50 μm. Data are presented as the mean ± SD (n=20; two-way ANOVA; ***p < 0.001)). The experimentswere repeated three times. **B**. The expression of γ-H2AX in DTX3 overexpression or not cells after IR was analyzed by western blotting. **C.** Representative fluorescence images and quantification of γ-H2AX foci immunostaining in HeLa cells with/without DTX3 overexpression. Above: representative fluorescence images (scale bars, 10 µm; red, γ-H2AX; blue, DAPI). Under: the percentage of positive cells with γ-H2AX foci (>10). Each datum point contains at least 20 cells. Data are presented as the mean ± SD (n=3 independent experiments; two-way ANOVA; ***p < 0.001). **D, E**. Characterization of DSB repair pathways by HRR (**D**) and non-homologous end joining (NHEJ) (**E**) reporter assays. The proportion of GFP^+^ cells was measured by flow cytometry and the efficiency was normalized to the control cells. Data are presented as mean ± SD (n = 3 independent experiments; unpaired t-test; ns, not significant; **p < 0.01). **F**. HeLa cells with/without DTX3 knockdown were collected at the indicated time after 6 Gy IR for the neutral comet assay. CaspLab-Comet Assay software was used to analyze the tail moment, , scale bar: 50 μm. Data are presented as the mean ± SD (n=20; two-way ANOVA; ***p < 0.001) The experimentswere repeated three times. **G**. The expression of γ-H2AX in DTX3 knockdown or not cells after IR was analyzed by western blotting. **H**. Representative fluorescence images and quantification of γ-H2AX foci immunostaining in HeLa cells with/without DTX3 knockdown. Above: representative fluorescence images (scale bars, 10 µm; red, γ-H2AX; blue, DAPI). Under: the percentage of positive cells with γ-H2AX foci (>10). Each datum point contains at least 20 cells. Data are presented as the mean ± SD (n=3 independent experiments; two-way ANOVA; ***p < 0.001). **I, J**. Characterization of DSB repair pathways by HRR (**I**) and NHEJ (**J**) reporter assays. The proportion of GFP^+^ cells was measured by flow cytometry and the efficiency was normalized to the control cells. Data are presented as mean ± SD (n = 3 independent experiments; one-way ANOVA; ns, not significant; ***p < 0.001). n=3 independent experiments. Abbreviations: IR, irradiation. The unprocessed images of the blots are shown in Figure S23 (Supporting Information).

Figure S3. Re-expression of DTX3 reverses the DTX3 knockdown-mediated promotion of clonogenic formation after irradiation. A. The DTX3 expression efficiency was detected by the western blotting assay in HK1 and HeLa cells. **B, C**. Clonogenic assays and survival fraction curves after exposure to different IR of the indicated HK1 (**B**) and HeLa (**C**) cells. All data are presented as the mean ± SD. n=3 independent experiments, extra sum-of-squares F test, ***p < 0.001. Abbreviations: IR, irradiation. The unprocessed images of the blots are shown in Figure S23 (Supporting Information).

Figure S4. Re-expression of DTX3 reverses the inhibition of apoptosis caused by DTX3 knockdown. A, B. Flow cytometric analysis of apoptosis in the indicated HK1 (**A**) and HeLa (**B**) cells at 24 h after exposure to either no IR or 6 Gy IR. All data are presented as the mean ± SD. n=3 independent experiments, one-way ANOVA, ***p < 0.001. Abbreviations: IR, irradiation.

Figure S5. **Re-expression of DTX3 restores the promotion of HRR repair induced by DTX3 knockdown in HK1 and HeLa cells. A, B.** The activity of HRR- or NHEJ-mediated DNA repair was quantified as the fraction of GFP⁺ cells normalized to controls in HK1 (**A**) and HeLa (**B**) cells. The percentage of GFP^+^ cells was analyzed by flow cytometry. All data are presented as the mean ± SD. n=3 independent experiments, one-way ANOVA, *p < 0.05, ***p < 0.001.

Figure S6. **DTX3 binds to NPM3. A.** HA-DTX3 junction-specific peptides of TOP2A, TOP2B, PSMB2, and NPM3 were identified with immunoprecipitation of anti-HA antibody in HK1 cells stably overexpressing HA-DTX3. **B**. The exogenous interaction of DTX3 and NPM3 was demonstrated by Coimmunoprecipitation (Co-IP) in HeLa cells cotransfected with HA-DTX3 and Flag-NPM3. **C**. Co-IP assays confirmed the endogenous interaction between DTX3 and NPM3 in HK1 and HeLa cells. **D**. The interaction between DTX3 and NPM3 was unaffected over 24 h after 6 Gy irradiation. **E**. Immunofluorescence staining showed nuclear colocalization of exogenous HA-DTX3 (green) and endogenous NPM3 (red) at 6 h following exposure to 6 Gy in HeLa cells. Scale bars: 5 μm. **F.** Co-IP and immunoblot analysis of lysates from HeLa cells transfected with HA-DTX3 or its mutants and Flag-NPM3. **G**. Co-IP and immunoblot analysis of lysates from HeLa cells transfected with Flag-NPM3 or its mutants and HA-DTX3. **H**. HeLa cells were transfected with *DTX3, vector, shNC or shDTX3#1* to detect the expressions of NPM3 after IR with 6Gy for 6 h. n=3 independent experiments. Abbreviations: IR, irradiation. The unprocessed images of the blots are shown in Figure S23(Supporting Information).

**Figure** S7. DTX3 ubiquitinates and degrades NPM3. **A, B.** Effects of cycloheximide (CHX) treatment transfected with Flag-*NPM3* and HA-*DTX3* or empty vector plasmids (A), as well as in shNC or sh*DTX3#1* (B) in HeLa cells. Data are presented as the mean ± SD (n=3 independent experiments; two-way ANOVA; **p < 0.01). **C, D**. The results of treatment with MG132 and Chloroquine (CQ) transfected with Flag-*NPM3* and HA-*DTX3* or empty vector plasmids (**C**), as well as in shNC or sh*DTX3#1* (**D**) in HeLa cells. **E**. HeLa cells were transfected with *DTX3* or vector for 36 h and treated with MG132 for another 6 h before western blot analyses. **F**. HK1 cells expressing sh*DTX3#1* or the shNC were transfected with Flag-*NPM3* and HA-*WT-Ub* or its mutants (HA-*K6O-Ub,* HA-*K11O-Ub,* HA-*K27O-Ub,* HA-*K29O-Ub,* HA-*K33O-Ub,* HA-*K48O-Ub* or HA-*K63O-Ub*), and were treated with MG132. Western blotting images of denature-IP experiments. **G**. HK1 cells expressing sh*DTX3#1* or shNC were cotransfected with HA-*Ub(K27O)*, and Flag-*NPM3* WT or KR mutants for denature-IP. n=3 independent experiments. Abbreviations: IR, irradiation. The unprocessed images of the blots are shown in Figure S23 (Supporting Information).

**Figure** S8. **NPM3 binds to RUVBL1 and inhibits 53BP1 binding at DSB sites.** A. Flag-NPM3 junction-specific peptides of RUVBL1 were identified with immunoprecipitation of anti-Flag antibody in HK1 cells stably overexpressing Flag-NPM3. **B**. The interaction between NPM3 and RUVBL1 was unaffected over 24 h after 6 Gy irradiation. **C**. GST pull-down assay indicating the direct interaction of purified GST-NPM3 protein with Myc-RUVBL1 protein. **D**. Immunofluorescence staining showed nuclear colocalization of exogenous Flag-NPM3 (green) and endogenous RUVBL1 (red) at 6 h following exposure to 6 Gy in HeLa cells. Scale bars: 5 μm. **E**. The HK1 cells in which NPM3 was knocked down or not were irradiated with 6Gy and analyzed using immunoblot with H4K16Ac, RUVBL1, and NPM3 antibodies. **F**. HeLa cells transfected with the indicated plasmids were irradiated with 6Gy, then collected and incubated with the anti-53BP1 antibody. Left: representative fluorescence images (scale bars, 10 µm; red, 53BP1; blue, DAPI). Right: the percentage of positive cells with 53BP1 foci (>10). Each datum point contains at least 20 cells. Data are presented as the mean ± SD (n=3 independent experiments; two-way ANOVA; *p < 0.05, **p < 0.01, ***p < 0.001). **G**. Representative confocal images for 53BP1 and γ-H2AX in HK1 and HeLa cells transiently transfected with DTX3 and treated with IR (6 Gy) and immunostained after 6 h (left). Co-localization was confirmed with both fluorescent intensity profiles and Pearson’s correlation coefficient determination. Fluorescence-intensity profiles of 53BP1 and γ-H2AX were obtained using the Leica LAS AF software, along a straight line (white) crossing the nucleus of a representative cell (right). Abbreviations: IR, irradiation; D&N, DTX3+NPM3. The unprocessed images of the blots are shown in Figure S23 (Supporting Information).

**Figure** S9. The GESA analysis on NPM3 and t**he expression efficiency of cancer cells transfected with the indicated plasmids.** **A.** GSEA of the GSE12452 dataset revealed enrichment of UV response and DNA damage response pathways in the NPM3 high-expression group. **B, C.** Western blotting and RT-qPCR analysis of the cells with Flag-NPM3 overexpression in HK1 and HeLa cells. **D, E**. Western blotting and RT-qPCR analysis of the cells with NPM3 knockdown in HK1 and HeLa cells. All data are presented as the mean ± SD. n=3 independent experiments, unpaired t-test, ***p < 0.001. The unprocessed images of the blots are shown in Figure S23 (Supporting Information).

**Figure** S10. **NPM3 knockdown decreases radioresistance in HeLa cells in vitro. A, B.** Clonogenic assays and survival fraction curves after exposure to indicated IR dose(**A**), and CCK-8 assays after exposure to 6 Gy (**B**) of indicated HeLa cells. Data are presented as the mean ± SD (n=3; extra sum-of-squares F test was used for **A**, two-way ANOVA was used for **B**; *p < 0.05, ***p < 0.001). **C**. Flow cytometric analysis of apoptosis in HeLa cells with NPM3 knockdown or not at 24 h after exposure to either no IR or 6 Gy IR. Data are presented as the mean ± SD (n=3; one-way ANOVA; ***p < 0.001). n=3 independent experiments. Abbreviations: IR, irradiation.

**Figure** S11. **NPM3 overexpression promotes radioresistance in HK1 and HeLa cells in vitro.** **A, B.** Clonogenic assays and survival fraction curves after exposure to indicated IR dose(**A**), and CCK-8 assays after exposure to 6 Gy (**B**) of indicated HK1 cells. Data are presented as the mean ± SD (n=3; extra sum-of-squares F test was used for **A**, two-way ANOVA was used for **B**; *p < 0.05, ***p < 0.001). **C**. Flow cytometric analysis of apoptosis in HK1 cells with NPM3 overexpression or not at 24 h after exposure to either no IR or 6 Gy IR. Data are presented as the mean ± SD (n=3; unpaired t-test; ***p < 0.001). **D, E.** Clonogenic assays and survival fraction curves after exposure to indicated IR dose(**D**), and CCK-8 assays after exposure to 6 Gy (**E**) of indicated HeLa cells. Data are presented as the mean ± SD (n=3; extra sum-of-squares F test was used for **D**, two-way ANOVA was used for **E**; *p < 0.05, ***p < 0.001). **F**. Flow cytometric analysis of apoptosis in HeLa cells with NPM3 overexpression or not at 24 h after exposure to either no IR or 6 Gy IR. Data are presented as the mean ± SD (n=3; unpaired t-test; ***p < 0.001). n=3 independent experiments. Abbreviations: IR, irradiation.

**Figure** S12. **NPM3 knockdown impaires HRR in HeLa cells. A**. Representative comet images and quantitative analysis of tail moments at different time points after the indicated cells were treated with 6Gy, scale bar: 50 μm. Data are presented as the mean ± SD (n=20; two-way ANOVA; ***p < 0.001). The experiments were repeated three times. **B**. The expression of γ-H2AX in NPM3 knockdown or not HeLa cells after IR was analyzed by western blotting. **C.** Representative fluorescence images and quantification of γ-H2AX foci immunostaining in HeLa cells with/without NPM3 knockdown. Above: representative fluorescence images (scale bars, 10 µm; red, γ-H2AX; blue, DAPI). Under: the percentage of positive cells with γ-H2AX foci (>10). Each datum point contains at least 20 cells. Data are presented as the mean ± SD (n=3 independent experiments; two-way ANOVA; ***p < 0.001). **D, E**. Characterization of DSB repair pathways by HRR (**D**) and NHEJ (**E**) reporter assays. The proportion of GFP^+^ cells was measured by flow cytometry and the efficiency was normalized to the control cells. (n = 3 independent experiments; one-way ANOVA; ns, not significant; ***p < 0.001). Abbreviations: IR, irradiation. The unprocessed images of the blots are shown in Figure S23 (Supporting Information).

**Figure** S13. **NPM3 overexpression promotes HRR in HK1 and HeLa cells.** **A**. Representative comet images and quantitative analysis of tail moments at different time points after the indicated HK1 cells were treated with 6Gy, scale bar: 50 μm. Data are presented as the mean ± SD (n=20; two-way ANOVA; ***p < 0.001). The experiments were repeated three times. **B**. The expression of γ-H2AX in NPM3 overexpression or not HK1 cells after IR was analyzed by western blotting. **C.** Representative fluorescence images and quantification of γ-H2AX foci immunostaining in HeLa cells with/without NPM3 overexpression. Above: representative fluorescence images (scale bars, 10 µm; red, γ-H2AX; blue, DAPI). Under: the percentage of positive cells with γ-H2AX foci (>10). Each datum point contains at least 20 cells. Data are presented as the mean ± SD (n=3 independent experiments; two-way ANOVA; *p < 0.05, ***p < 0.001). **D, E**. Characterization of DSB repair pathways by HRR (**D**) and NHEJ (**E**) reporter assays. The proportion of GFP^+^ HK1 cells was measured by flow cytometry and the efficiency was normalized to the control cells. Data are presented as the mean ± SD (n = 3 independent experiments; unpaired t-test; ns, not significant; ***p < 0.001). **F**. Representative comet images and quantitative analysis of tail moments at different time points after the indicated HeLa cells were treated with 6Gy, scale bar: 50 μm. Data are presented as the mean ± SD (n=20; two-way ANOVA; ***p < 0.001). The experiments were repeated three times. **G**. The expression of γ-H2AX in NPM3 overexpression or not HeLa cells after IR was analyzed by western blotting. **H.** Representative fluorescence images and quantification of γ-H2AX foci immunostaining in HeLa cells with/without NPM3 overexpression. Above: representative fluorescence images (scale bars, 10 µm; red, γ-H2AX; blue, DAPI). Under: the percentage of positive cells with γ-H2AX foci (>10). Each datum point contains at least 20 cells. Data are presented as the mean ± SD (n=3 independent experiments; two-way ANOVA; ***p < 0.001). **I, J**. Characterization of DSB repair pathways by HRR (**I**) and NHEJ (**J**) reporter assays. The proportion of GFP^+^ HeLa cells was measured by flow cytometry and the efficiency was normalized to the control cells. Data are presented as the mean ± SD (n = 3 independent experiments; unpaired t-test; ns, not significant; ***p < 0.001). Abbreviations: IR, irradiation. The unprocessed images of the blots are shown in Figure S23 (Supporting Information).

**Figure** S14. Re-expression of NPM3 reverses the NPM3 knockdown-mediated promotion of clonogenic formation after irradiation. A. The NPM3 expression efficiency was detected by the western blotting assay in HK1 and HeLa cells. **B, C**. Clonogenic assays and survival fraction curves after exposure to different IR of the indicated HK1 (**B**) and HeLa (**C**) cells. Data are presented as the mean ± SD (n=3; extra sum-of-squares F test; ***p < 0.001). n=3 independent experiments. Abbreviations: IR, irradiation. The unprocessed images of the blots are shown in Figure S23 (Supporting Information).

Figure S15. Re-expression of NPM3 reverses the inhibition of apoptosis caused by NPM3 knockdown. A, B. Flow cytometric analysis of apoptosis in the indicated HK1 (**A**) and HeLa (**B**) cells at 24 h after exposure to either no IR or 6 Gy IR. Data are presented as the mean ± SD (n=3; one-way ANOVA; ***p < 0.001). n=3 independent experiments. Abbreviations: IR, irradiation.

Figure S16. **Re-expression of NPM3 restores the promotion of HRR repair induced by NPM3 knockdown in HK1 and HeLa cells. A, B.** The activity of HRR- or NHEJ-mediated DNA repair was quantified as the fraction of GFP⁺ cells normalized to controls in HK1 (**A**) and HeLa (**B**) cells. The percentage of GFP^+^ cells was analyzed by flow cytometry. Data are presented as the mean ± SD (n = 3 independent experiments; one-way ANOVA; ***p < 0.001).

**Figure** S17. **The VEC, *DTX3*, and *D&N* HK1 or HeLa cells were stably established.** The DTX3 and NPM3 expression efficiency was detected by the western blotting assay. n=3 independent experiments. Abbreviations: D&N, DTX3+NPM3. The unprocessed images of the blots are shown in Figure S23 (Supporting Information).

**Figure** S18. **Elevated NPM3 reverses the radiosensitizing effect of DTX3 in HeLa cells. A, B.** Clonogenic assays and survival fraction curves after exposure to indicated IR dose(**A**), and CCK-8 assays after exposure to 6 Gy (**B**) of indicated HeLa cells. Data are presented as the mean ± SD (n=3; extra sum-of-squares F test was used for **A**, two-way ANOVA was used for **B**; *p < 0.05, **p < 0.01, ***p < 0.001). **C**. Flow cytometric analysis of apoptosis in the indicated HeLa cells at 24 h after exposure to either no IR or 6 Gy IR. Data are presented as the mean ± SD (n=3; one-way ANOVA; ***p < 0.001). n=3 independent experiments. Abbreviations: IR, irradiation; D&N, DTX3+NPM3.

**Figure** S19. **Flow cytometric analysis of apoptosis in the indicated HK1 cells at 24 h after exposure to either no IR or 6 Gy irradiation.** Data are presented as the mean ± SD (n=3; one-way ANOVA; ***p < 0.001). Abbreviations: IR, irradiation.

**Figure** S20. **Elevated NPM3 reverses DTX3 mediated inhibition of HRR in HeLa cells. A.** Representative comet images and quantitative analysis of tail moments at different time points after the HeLa cells were treated with 6Gy, scale bar: 50 μm. Data are presented as the mean ± SD (n=20; two-way ANOVA; ***p < 0.001)). The experiments were repeated three times. **B**. The expression of γ-H2AX in indicated cells after IR was analyzed by western blotting. **C.** Representative fluorescence images and quantification of γ-H2AX foci immunostaining in indicated HeLa cells. Above: representative fluorescence images (scale bars, 10 µm; red, γ-H2AX; blue, DAPI). Under: the percentage of positive cells with γ-H2AX foci (>10). Each datum point contains at least 20 cells. Data are presented as the mean ± SD (n=3 independent experiments; two-way ANOVA; ***p < 0.001). **D, E**. Characterization of DSB repair pathways by HRR (**D**) and NHEJ (**E**) reporter assays. The proportion of GFP^+^ cells was measured by flow cytometry and the efficiency was normalized to the control cells. Data are presented as mean ± SD (n = 3 independent experiments; one-way ANOVA; ns, not significant; ***p < 0.001). n=3 independent experiments. Abbreviations: IR, irradiation; D&N, DTX3+NPM3. The unprocessed images of the blots are shown in Figure S23 (Supporting Information).

**Figure** S21. Lower expression of DTX3 is associated with poor prognosis. A, B, C. Forest plots of multivariate Cox regression analyses showing the significance of different prognostic variables in nasopharyngeal carcinoma locoregional recurrence-free survival (LRFS, A), disease-free survival (DFS, B), and overall survival (OS, **C**). D, E. Kaplan–Meier analysis of OS, and progression-free survival (PFS) according to DTX3 expression levels using the log-rank test in TCGA-LGG (**D**) and TCGA-GBMLGG (**E**) dataset. Abbreviations: LGG, low grade glioma; GBMLGG, diffuse glioma.

**Figure** S22. **Low expression of DTX3 correlates with unfavorable radiotherapy （****RT）prognosis.** A. Kaplan–Meier analysis of OS according to DTX3 expression in TCGA-BLCA dataset of patients with or not radiotherapy. The 32nd percentile was used as the cut-off point. **B**. Kaplan-Meier analysis of OS for the 16th, 32nd, 48th percentiles of DTX3 expression in TCGA-BLCA dataset. **C**. Kaplan–Meier analysis of OS according to DTX3 expression in TCGA-UCS dataset of patients with or not radiotherapy. The 42nd percentile was used as the cut-off point. **D**. Kaplan-Meier analysis of OS for the 21st, 42nd, 63rd percentiles of DTX3 expression in TCGA-UCS dataset. **E**. Kaplan–Meier analysis of OS according to DTX3 expression in TCGA-LUAD dataset of patients with or not radiotherapy. The lower-quartile was used as the cut-off point. **F**. Kaplan-Meier analysis of OS for the 25th, 50th, 75th percentiles of DTX3 expression in TCGA-LUAD dataset. **G**. Kaplan–Meier analysis of PFS according to DTX3 expression in TCGA-LUAD dataset of patients with or not radiotherapy. The lower-quartile and median was used as the cut-off point. **H**. Kaplan-Meier analysis of PFS for the 25th, 50th, 75th percentiles of DTX3 expression in TCGA-LUAD dataset. Data in B, D, F, H were presented as forest plots based on Hazard Ratio (HR) and 95% confidence interval. Red dashed line indicated HR=1. The p values were determined using the log-rank test. All p-value< 0.05 as statistic difference. Abbreviations: BLCA, bladder urothelial carcinoma; UCS, uterine carvinosarcoma; LUAD, lung adenocarcinoma.

Figure S23. (Unprocessed immunoblots)

Unprocessed immunoblots for indicated Figures panels.

Figure S23. (Unprocessed immunoblots)

Unprocessed immunoblots for indicated Figures panels.

Figure S23. (Unprocessed immunoblots)

Unprocessed immunoblots for indicated Figures panels.

Figure S23. (Unprocessed immunoblots)

Unprocessed immunoblots for indicated Figures panels.

Figure S23. (Unprocessed immunoblots)

Unprocessed immunoblots for indicated Figures panels.

Figure S23. (Unprocessed immunoblots)

Unprocessed immunoblots for indicated Figures panels.

Figure S23. (Unprocessed immunoblots)

Unprocessed immunoblots for indicated Figures panels.

**Table S1. List of E3 ligase library (n=769)**

**Table S2**. **List of protein candidates that interact with HA‑DTX3 identified by mass spectrometry.**

**For the band with MW ≈180 kDa.**

**For the band with MW ≈25 kDa.**

**Table S3**. **List of protein candidates that interact with Flag-NPM3 identified by mass spectrometry.**

**Table S4**. **Correlations between DTX3 expression levels and clinical features in patients with nasopharyngeal carcinoma.**

|  | **Low expression** | **High expression** | **p-value** |
| --- | --- | --- | --- |
|  | **(N=97)** | **(N=96)** |  |
| **Gender** |  |  | 0.353 |
| male | 68 (70.1%) | 73 (76.0%) |  |
| female | 29 (29.9%) | 23 (24.0%) |  |
| **Age** |  |  | 0.720 |
| ≤45 | 46 (47.4%) | 48 (50.0%) |  |
| >45 | 51 (52.6%) | 48 (50.0%) |  |
| **WHO type** |  |  | 0.431 |
| Ⅰ | 1 (1.0%) | 0 (0%) |  |
| Ⅱ | 2 (2.1%) | 4 (4.2%) |  |
| Ⅲ | 94 (96.9%) | 92 (95.8%) |  |
| **TNM stage** |  |  | 0.889 |
| Ⅰ | 3 (3.1%) | 2 (2.1%) |  |
| Ⅱ | 20 (20.6%) | 17 (17.7%) |  |
| Ⅲ | 43 (44.3%) | 47 (49.0%) |  |
| Ⅳ | 31 (32.0%) | 30 (31.3%) |  |
| **VCA-IgA** |  |  | 0.589 |
| ≤1:80 | 18 (18.6%) | 15 (15.6%) |  |
| >1:80 | 79 (81.4%) | 81 (84.4%) |  |
| **EA-IgA** |  |  | 0.250 |
| ≤1:10 | 25 (25.8%) | 32 (33.3%) |  |
| >1:10 | 72 (74.2%) | 64 (66.7%) |  |
| **Death** |  |  | **0.037** |
| No | 58 (59.8%) | 71 (74.0%) |  |
| Yes | 39 (40.2%) | 25 (26.0%) |  |
| **Disease** |  |  | **0.029** |
| No | 54 (55.7%) | 68 (70.8%) |  |
| Yes | 43 (44.3%) | 28 (29.2%) |  |
| **Locoregional recurrence** |  |  | **0.020** |
| No | 84 (86.6%) | 93 (96.9%) |  |
| Yes | 13 (13.4%) | 3 (3.1%) |  |

The p-values were determined using the two-tailed χ2 test. Abbreviations: WHO, World Health Organization; VCA-IgA, viral capsid antigen immunoglobulin A; EA-IgA, early antigen immunoglobulin A.

**Table S5**. **Clinicopathological data of 193 paraffin-embedded NPC tissues for immunohistochemistry staining.**

**Table S6. List of primers and shRNA sequences used in this study.**

| **Gene** | **Sequence (5’ to 3’)** |
| --- | --- |
| **Real-time RT-PCR primers** |  |
| DTX3-F | CCAGCGTCTCACCTTCACTATCG |
| DTX3-R | TGGTCTTGTGGTGGATGTCGTTC |
| NPM3-F | GATGACTTCCAGCTCCAACCA |
| NPM3-R | CTCGCTCTCCTCCTCAGAAACA |
| GAPDH-F | TGATGACATCAAGAAGGTGG |
| GAPDH-R | TTGTCATACCAGGAAATGAGC |
| **shRNA sequences** |  |
| shRNA-DTX3#1 | CCGGCGGGACCATCCTCATAGTTTACTCGAGTAAACTATGAGGATGGTCCCGTTTTTG |
| shRNA-DTX3#2 | CCGGCAGAGATGGACCGCAACATTACTCGAGTAATGTTGCGGTCCATCTCTGTTTTTG |
| shRNA-NPM3#1 | CCGGCATGCTCAGTCTGGATGACTTCTCGAGAAGTCATCCAGACTGAGCATGTTTTTG |
| shRNA-NPM3#2 | CCGGCGAGTGTAATGTGGTAGAAGTCTCGAGACTTCTACCACATTACACTCGTTTTTG |

**Table S7. List of antibodies used in this study.**

| **Antibody** | **Company** | **Catalog no.** | **Dilution** |
| --- | --- | --- | --- |
| **Western blot** |  |  |  |
| β-Actin antibody, Rabbit Polyclonal | CST, USA | 4970S | 1:1000 |
| GAPDH antibody, Mouse Monoclonal | Proteintech, USA | 60004-1- -Ig | 1:100000 |
| Anti-DTX3 antibody, Rabbit Polyclonal | Proteintech, USA | 25304-1-AP | 1:5000 |
| Anti-TOP2A antibody, Mouse Monoclonal | Proteintech, USA | 20233-1-AP | 1:5000 |
| Anti-TOP2B antibody, Rabbit Polyclonal | Proteintech, USA | 20549-1-AP | 1:1000 |
| Anti-PSMB2 antibody, Rabbit Polyclonal | Proteintech, USA | 15154-1-AP | 1:1000 |
| Anti-NPM3 antibody, Rabbit Polyclonal | Proteintech, USA | 11960-1-AP | 1:4000 |
| Anti-HA antibody produced in rabbit | Sigma-Aldrich, USA | H6908 | 1:1000 |
| Anti-Flag antibody produced in mouse | Sigma-Aldrich, USA | F1804 | 1:500 |
| Myc-Tag Antibody, Rabbit Polyclonal | Proteintech, USA | 16286-1-AP | 1:2000 |
| Phospho-Histone H2A.X (Ser139) (20E3), Rabbit mAb | CST, USA | 9718S | 1:1000 |
| Anti-RUVBL1 antibody, Rabbit Polyclonal | Proteintech, USA | 10210-2-AP | 1:2000 |
| Anti-H4K16Ac antibody, Rabbit Polyclonal | Zenbio | R380899 | 1:1500 |
| Anti-TIP60 antibody, Mouse Monoclonal | Zenbio | 221268 | 1:700 |
| GST-Tag Monoclonal antibody | Proteintech, USA | 66001 | 1:20000 |
| Anti-rabbit IgG, HRP-linked Antibody | CST, USA | 7074S | 1:5000 |
| **Immunohistochemistry staining** |  |  |  |
| Anti-DTX3 antibody, Mouse Monoclonal | Santa Cruz Biotechnology, USA | Sc-376439 | 1:50 |
| **Immunofluorescence** |  |  |  |
| Anti-NPM3 antibody, Rabbit Polyclonal | Proteintech, USA | 11960-1-AP | 1:100 |
| Anti-RUVBL1 antibody, Rabbit Polyclonal | Proteintech, USA | 10210-2-AP | 1:100 |
| Phospho-Histone H2A.X (Ser139) (20E3), Rabbit mAb | CST, USA | 9718S | 1:400 |
| Anti-53BP1 antibody, Mouse Monoclonal | Sigma-Aldrich, USA | MAB3802 | 1:500 |
| Anti-HA antibody produced in rabbit | Sigma-Aldrich, USA | H6908 | 1:400 |
| Anti-HA antibody produced in mouse | Sigma-Aldrich, USA | H3663 | 1:400 |
| Anti-Flag antibody produced in rabbit | Proteintech, USA | 20543-1-AP | 1:400 |
| Cy3–conjugated Rabbit Anti-Goat IgG(H+L) | Proteintech, USA | SA00009-4 | 1:50 |
| Fluorescein (FITC)–conjugated Goat Anti-Mouse IgG(H+L) | Proteintech, USA | SA00003-1 | 1:50 |
| **Co-Immunoprecipitation** |  |  |  |
| Anti-HA antibody produced in rabbit | Sigma-Aldrich, USA | H6908 | 3µg |
| Anti-Flag antibody produced in mouse | Sigma-Aldrich, USA | F1804 | 3µg |
| Anti-RUVBL1 antibody, Rabbit Polyclonal | Proteintech, USA | 10210-2-AP | 3µg |
| Normal Mouse IgG | Invitrogen, USA | 10400C | 3µg |
| Normal Rabbit IgG | Invitrogen, USA | 10500C | 3µg |
| Anti-NPM3 antibody, Rabbit Polyclonal | Proteintech, USA | 11960-1-AP | 3µg |
| **GST pull-down** |  |  |  |
| GST-Tag Monoclonal antibody | Proteintech, USA | 66001 | 1:20000 |
| Anti-DTX3 antibody, Rabbit Polyclonal | Proteintech, USA | 25304-1-AP | 1:5000 |
| Myc-Tag Antibody, Rabbit Polyclonal | Proteintech, USA | 16286-1-AP | 1:2000 |
